# A Review of Designing Hierarchical Structure Within Membrane Electrode Assembly for Water Electrolyzer

**DOI:** 10.1002/advs.202510546

**Published:** 2025-08-21

**Authors:** Saifei Pan, Yongqi Ye, Chuyu Zhang, Xin Chen, Xuetao Wang, Chunmei Liu, Haojie Li, Bin Tian, Fang Wang, Zongkui Kou

**Affiliations:** ^1^ School of Vehicle and Transportation Engineering Henan University of Science and Technology Luoyang 471000 China; ^2^ Division of Advanced Nanomaterials, Suzhou Institute of Nano‐Tech and Nano‐Bionics Chinese Academy of Sciences (CAS) Suzhou 215123 China; ^3^ School of Environmental Engineering and Chemistry Luoyang Institute of Science and Technology Luoyang Henan 471023 China; ^4^ State Key Laboratory of Advanced Technology for Materials Synthesis and Processing Wuhan University of Technology Wuhan Hubei 430070 China

**Keywords:** AEM/PEM water electrolyzers, enhancement mechanism, fabrication methods, hierarchical structure, membrane electrode assembly

## Abstract

Green hydrogen produced from water electrolyzers demonstrates higher efficiency and sustainability than industrial alkaline water electrolysis due to the membrane electrode assembly (MEA) design. However, random structure designs in current MEAs significantly increase the charge and mass transport resistance, leading to a decrease in energy efficiency. In contrast, the ordered structure design in MEA provides well‐defined arrangements of pores, channels, or pathways within catalysts, catalyst layers, porous transport layers, and ion exchange membranes (IEMs). These ordered configurations facilitate efficient pathways for charge and mass transport. Particularly, in comparison with first‐order structure, hierarchical structure designs exhibit more obvious advantages in reaction interface, charge, and mass transport. Recently, the diverse hierarchical structure in the MEA designs has demonstrated significant improvements in overall electrolysis efficiency in both proton exchange membrane (PEM) and anion exchange membrane (AEM) water electrolyzers. This review will examine recent advancements in hierarchical structure designs in the MEAs for water electrolyzers, focusing on innovations in fabrication methods and enhancement mechanisms, as well as their electrolysis performance. This review will provide comprehensive guidelines for designing highly efficient MEAs for both PEM and AEM electrolyzers.

## Graphical abstract

This review systematically summarizes recent advances in hierarchical structure designs within membrane electrode assemblies (MEAs) for both proton exchange membrane and anion exchange membrane water electrolyzers. Emphasis is placed on the structural evolution from disordered to ordered and hierarchical architectures, their impact on charge and mass transport, interfacial reactions, and electrolysis efficiency. Representative fabrication strategies and mechanistic insights are also discussed to provide a comprehensive framework for the rational design of high‐performance MEAs.

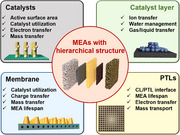

## 1 Introduction

Hydrogen energy is regarded as an ideal energy carrier in the 21^st^ century due to its high energy density (140 MJ kg^−1^) and complete environmental friendliness.^[^ ^1^, ^2^, ^3^, ^4^, ^5^ ^]^ However, large‐scale production of hydrogen through a green route remains a significant challenge. Compared to conventional alkaline water electrolysis (ALK), proton exchange membrane (PEM) and anion exchange membrane (AEM) water electrolysis are considered superior methods to produce hydrogen due to their higher efficiency, greater hydrogen purity, and wider dynamic operation range.^[^ ^6^, ^7^ ^]^ As the core component of AEM/PEM water electrolyzers, the membrane electrode assembly (MEA) is the site where multiphase transport and the hydrogen/oxygen evolution reactions occur, directly determining the electrolysis efficiency, lifespan, and cost of the system. Therefore, the design of high‐performance MEAs has received great attention in recent years.

**Figure 1:**
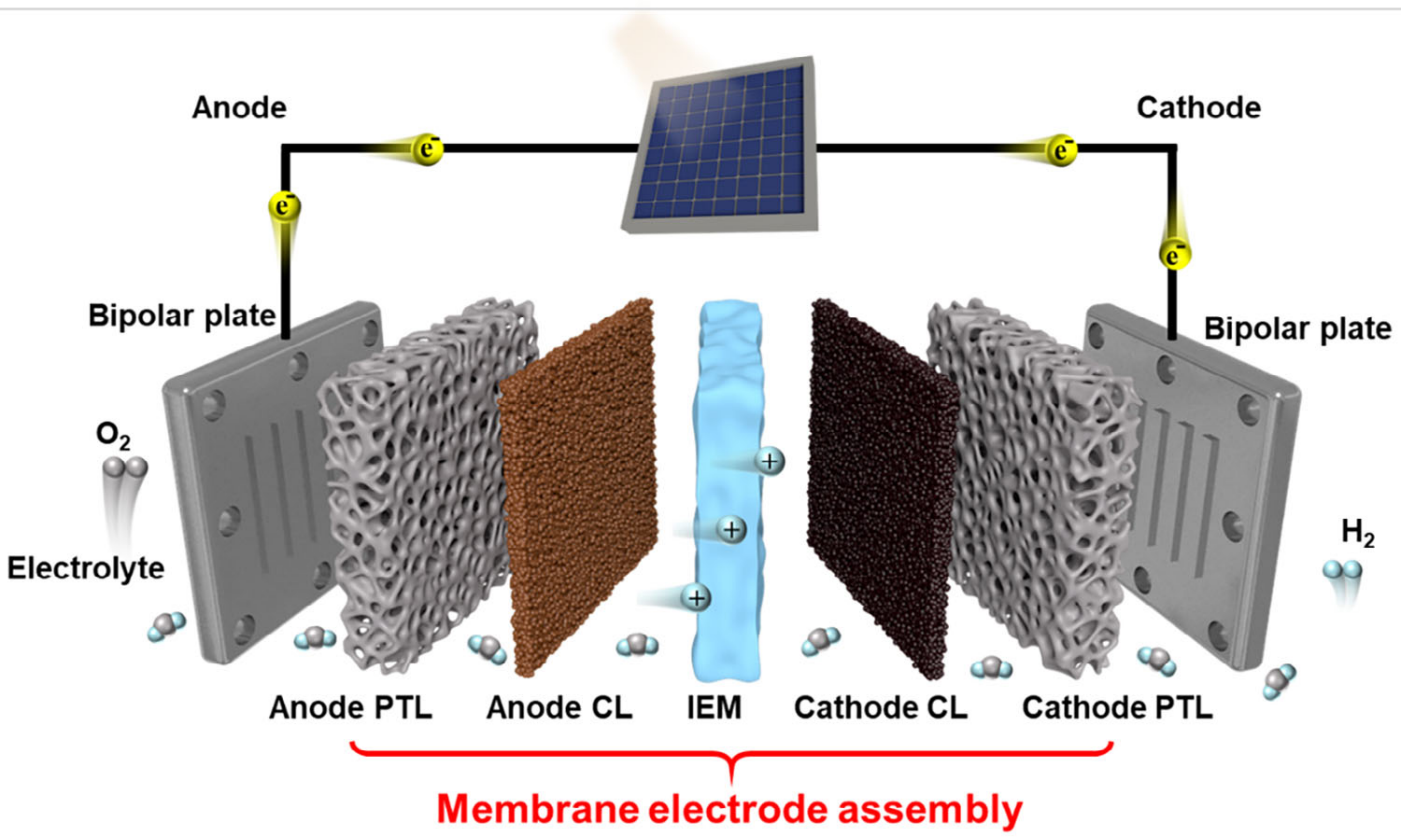
Schematic diagram of PEM/AEM water electrolyzers based on membrane electrode assembly (MEA), CL: catalyst layer, PTL: porous transport layer, IEM: ion exchange membrane.

**Figure 2:**
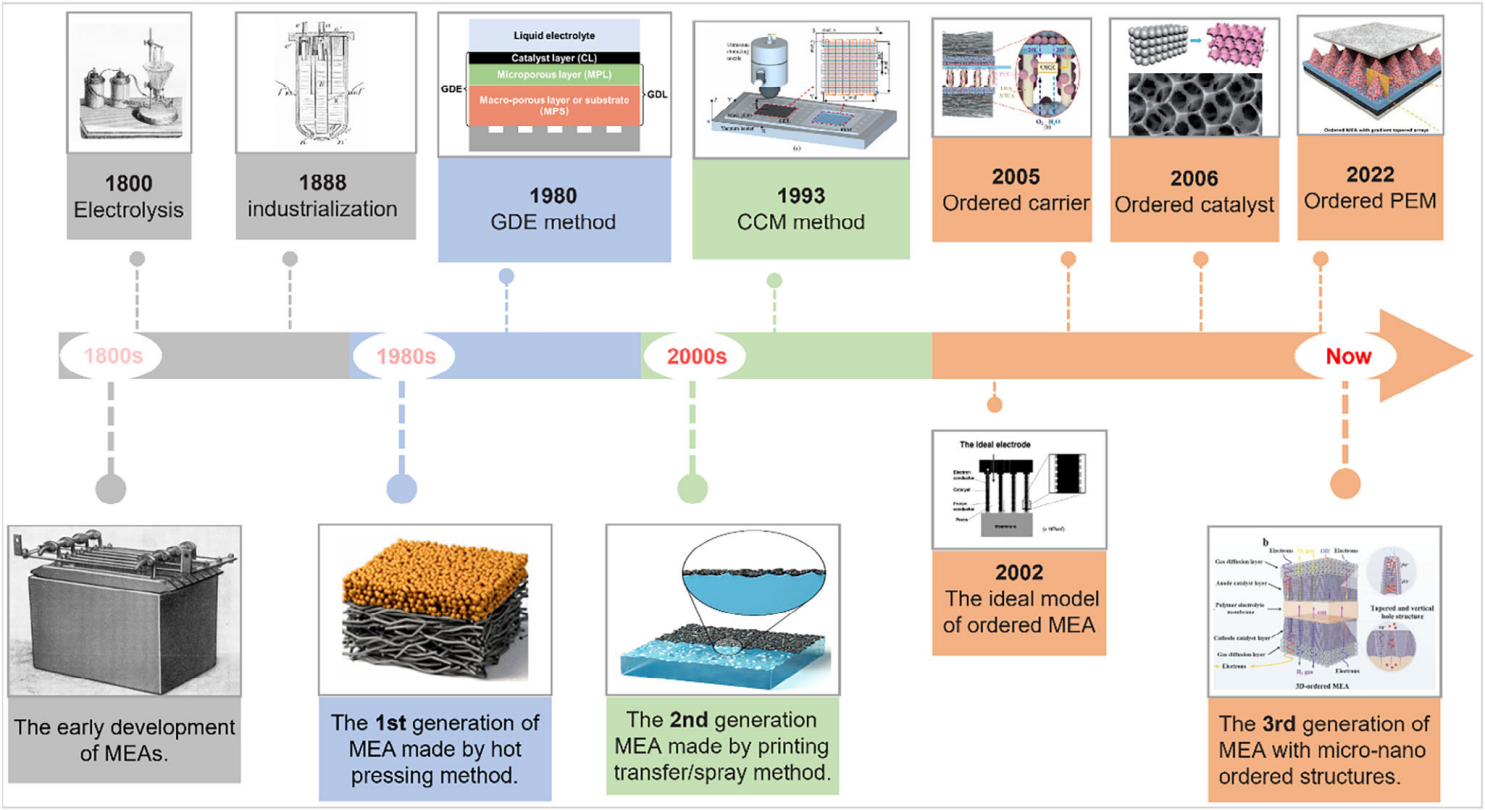
Timeline of major developments of MEAs in the application of ion exchange membrane water electrolysis. 1800s: Reproduced with permission. ^[^ ^17^ ^]^ Copyright 2022, Elsevier. 1980s: Reproduced with permission.^[^ ^18^ ^]^ Copyright 2021, American Chemical Society. 2000s: Reproduced with permission.^[^ ^19^ ^]^ Copyright 2022, American Chemical Society. 1993 and 2005: Reproduced with permission.^[^ ^20^ ^]^ Copyright 2023, Elsevier. 2002: Reproduced with permission. ^[^ ^21^ ^]^ Copyright 2002, Elsevier. 2022: Reproduced with permission.^[^ ^22^ ^]^ Copyright 2022, American Chemical Society. 2006: Reproduced with permission.^[^ ^23^ ^]^ Copyright 2014, Royal Society of Chemistry. The 3rd generation of MEA: Reproduced with permission.^[^ ^24^ ^]^ Copyright 2022, Royal Society of Chemistry.

Since Nicholson and Carlisle discovered that electricity can split water into H_2_ and O_2_, water electrolysis has attracted much attention. As shown in **Figure** **1**, a membrane electrode assembly (MEA) consists of five closely integrated components: the ion exchange membrane (IEM), cathode/anode catalyst layers (CLs), and anode/cathode porous transport layers (PTLs), forming a sandwich structure. To date, membrane electrode assemblies (MEAs) have undergone several stages, as shown in **Figure** **2**. The first‐generation MEAs are constructed using catalyst‐coated gas diffusion electrodes (GDEs). These MEAs faced several significant limitations, including high interfacial resistance and mechanical instability, thus hindering their performance and commercial viability.^[^ ^8^, ^9^, ^10^ ^]^ With this in mind, second‐generation MEAs have efficiently solved these issues by coating catalysts on the IEM surface (Figure 2). However, the stack configuration of the catalyst wrapped by ionomers not only gives rise to low catalyst utilization but also extends the transport path of charges, reactants, and products.^[^ ^11^, ^12^ ^]^ Afterwards, Hua et al. proposed the ideal model of ordered MEA, which offers several key advantages over previous generations, addressing many of the shortcomings seen in second‐generation designs,^[^ ^13^, ^14^, ^15^, ^16^ ^]^ including higher catalyst utilization, lower charge and mass transport resistance, as well as greater ion conductivity.

**Figure 3:**
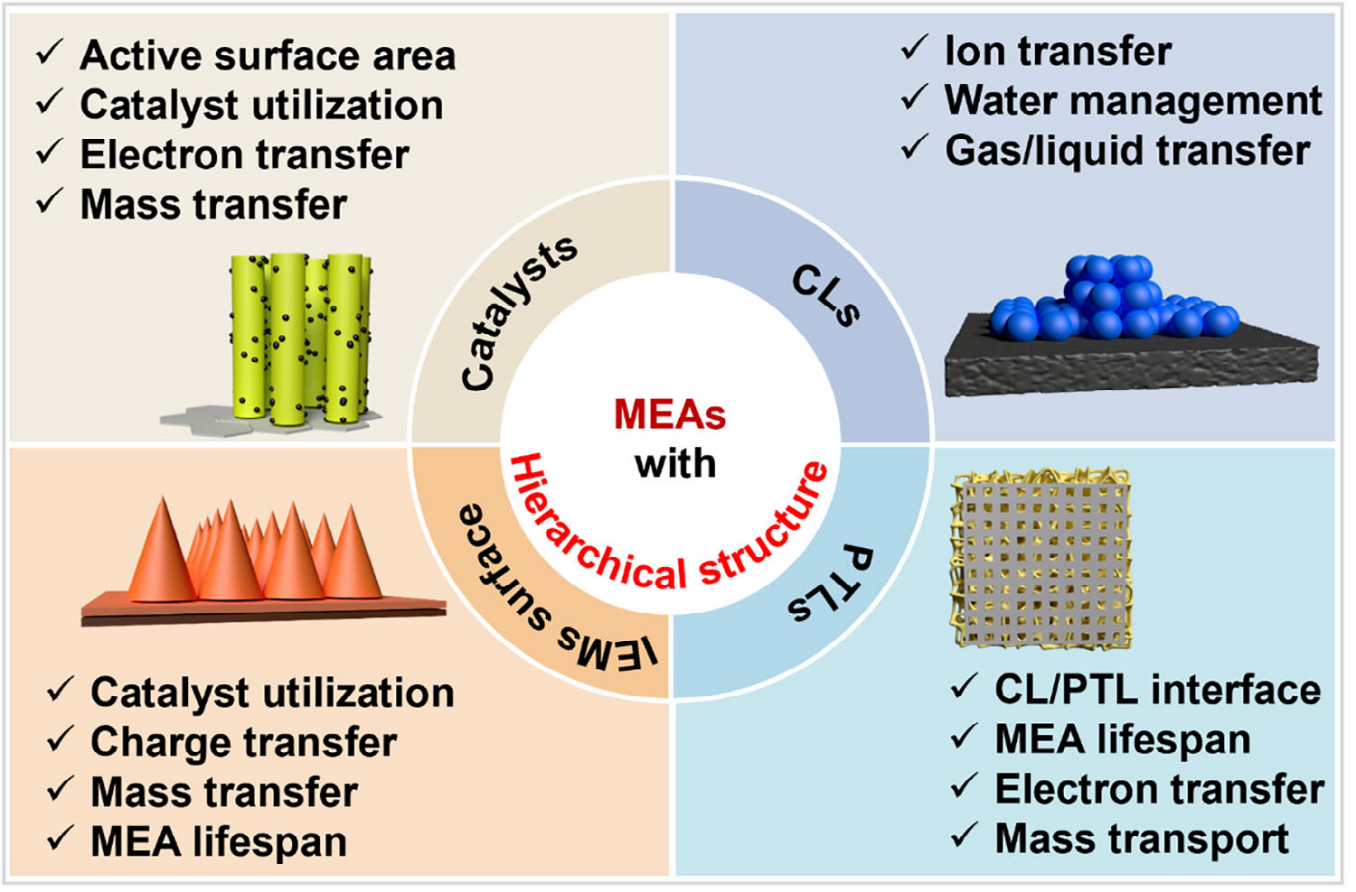
The main content of this review.

In comparison with first‐order structures, hierarchical structure designs exhibit more significant advantages in reaction interfaces, as well as in charge and mass transport.^[^ ^25^, ^26^, ^27^, ^28^ ^]^ In general, the hierarchical structures in the MEAs can be divided into four main types (**Figure** **3**): hierarchically structured catalysts and CL, IEM with hierarchical surface structures, and multi‐level structured PTLs. The ordered hierarchical structure designs improve the performance of MEAs in various aspects. In particular, catalysts with a hierarchical structure offer a larger surface area compared to first‐order structures, allowing for more active sites for electrochemical reactions.^[^ ^29^, ^30^ ^]^ This boosts overall water management efficiency and current density. The multi‐level structured catalyst layer facilitates better diffusion of reactants and products, as well as improved electron transfer, which reduces transport resistance, enhances ion conductivity, and accelerates reaction kinetics.^[^ ^31^, ^32^ ^]^ The hierarchical surface structure on the IEM enhances the membrane's mechanical strength, making it more resistant to deformation, delamination, or degradation during prolonged use.^[^ ^33^, ^34^ ^]^ Furthermore, it increases the reaction interfaces with the catalyst layers, thus significantly improving ions conductivity in the MEA. The PTLs with a multi‐level structure have been demonstrated to be beneficial for both gas and liquid transport.^[^ ^35^, ^36^, ^37^ ^]^

In recent years, considerable progress has been made in advancing catalyst nanostructures and MEA components at different scales. In particular, early work has clarified the relationship between hierarchical catalyst morphology and activity, primarily at the nanoscale.^[^ ^38^ ^]^ Subsequent studies have explored electrode porosity and catalyst dispersion with a focus on optimizing anode‐cathode interfaces.^[^ ^39^ ^]^ Important insights into ionomer distribution and its impact on catalyst‐ionomer interactions have further informed MEA design strategies. Recent developments in scalable fabrication and patterning techniques have opened new possibilities for catalyst layer structuring,^[^ ^40^, ^41^ ^]^ while emerging methods such as nanoimprinting show promise in controlling porosity and alignment at the microscale.^[^ ^42^ ^]^ Collectively, these efforts deepen the understanding of individual MEA elements; however, integrating multi‐scale hierarchical design across the entire MEA remains a key challenge and opportunity for enhancing overall electrolyzer performance and durability. In contrast, our review provides a systematic and MEA‐centered framework, focusing on how hierarchical structures across different MEA components (catalyst structure, CL, PTL, and membrane) synergistically affect mass‐charge transport and interfacial properties. We also emphasize the concept of nested interfaces and conformal integration between sublayers, which has not been comprehensively covered in the above‐mentioned works. Furthermore, the relationship between the microstructure components of MEA and the performance of water electrolyzers remains unclear.

To bridge the above gap, this review comprehensively overviews the development and challenges of MEAs for AEM/PEM water electrolysis. First, we provide an outline of the basics of MEAs at the single‐cell level. Then, we summarize recent advances in designing hierarchical structures in the MEAs for AEM/PEM water electrolysis. Based on these microstructure designs, we review AEM/PEM water electrolysis test processes to highlight key factors for achieving high‐performance water electrolysis. Finally, we discuss overall design strategies for hierarchical structures aimed at high‐performance MEAs, including catalyst structures, CL structures, IEM surface structures, and PTL structures, and corresponding interface structures. We anticipate that this review will serve as a useful introduction to the fundamentals of AEM/PEM water electrolysis and provide inspiration for further designs of high‐performance MEAs to enable the large‐scale application of green hydrogen production.

## 2 Fundamentals of AEM/PEM water electrolysis

IEMs water electrolysis is a critical technology for green hydrogen production, facilitating the electrochemical splitting of water into hydrogen and oxygen. This process leverages ion‐conductive membranes to enable efficient ion transport while maintaining gas separation. It is a complex process involving ion, electron, and mass transport.^[^ ^43^ ^]^ The total process is described as follows:

(1) $$2H_{2}O\;\left(l\right)\to 2H_{2}\left(g\right)+O_{2}\left(g\right)$$

The two principal types of IEM water electrolysis systems are PEM and AEM electrolyzers, the corresponding electrode's reactions can be written as follows:

AEM water electrolysis:

(2) $$\mathrm{Cathode}:\;2H_{2}O\;\left(l\right)+2e^{-}\to H_{2}\left(g\right)+2OH^{-}\left(\mathrm{aq}\right)$$

(3) $$\mathrm{Anode}:\;4OH^{-}\left(\mathrm{aq}\right)\to 2H_{2}O\;\left(l\right)+O_{2}\left(g\right)+4e^{-}$$

PEM water electrolysis:

(4) $$\mathrm{Cathode}:\;2H^{+}\left(\mathrm{aq}\right)+2e^{-}\to H_{2}\left(g\right)$$

(5) $$\mathrm{Anode}:\;2H_{2}O\;\left(l\right)\to 4H^{+}\left(\mathrm{aq}\right)+O_{2}\left(g\right)+4e^{-}$$

IEMs water electrolysis operates based on the application of an external voltage to drive redox reactions at the electrodes. For standard conditions, it can be calculated as 1.23 V (*E*_std_). However, in practical IEMs water electrolysis, this is never accomplished since an extra overpotential is required to meet extra energy consumption (e.g., activation, ions diffusion, and ohm resistance). Therefore, the actual cell voltage (*E*_cell_) is obviously higher than that of *E*_std_, composed of multiple contributing elements. In particular, the water electrolysis process first requires an additional overpotential (*η*_kin_) to overcome the energy barrier of oxygen/hydrogen evolution reaction (OER/HER), which is mainly influenced by reaction kinetics, catalyst activity, and temperature. The value can be calculated based on the Tafel model, in which the Tafel slope *b* and exchange current density *i*_0_ are the governing kinetic parameters. The Tafel model was fitted to iR‐free alkaline water electrolysis cell voltages at low current densities. The entire *η*_kin_ of the cell is *η*_kin_ = b × log(*i*/*i*_0_).

Ohmic resistance is another factor that determines electrolysis efficiency, resulting from the resistance to the flow of ions through the electrolyte membrane and electrons through the electrodes and external circuit. This value is proportional to the current (*I*) and the internal resistance (*R*_ohm_) of the cell (*η*_ohm_ = *I* × *R*_ohm_). In which *R*_ohm_ is obtained from the high frequency resistance (HFR) in electrochemical impedance spectroscopy (EIS). Based on the equation, it can be concluded that the *η*_ohm_ will become a major factor influencing *E*_cell_ at a high current density. Furthermore, an additional overpotential (*η*_mass_) is required to drive the transport of reactants and products in the MEAs. This occurs due to limitations in the transport of reactants (e.g., H_2_O/OH^−^) to the reaction sites and the removal of products (e.g., H_2_/O_2_). This overpotential becomes significant at high current densities when reactant depletion or product accumulation affects reaction rates. *η*_mass_ is calculated by subtracting *E*_std_, *η*_kin_, and *η*_ohm_ from *E*_cell_. Therefore, the *E*_cell_ can be described as follows:

(6) $$E_{\mathrm{cell}}=E_{\mathrm{std}}+\eta_{\mathrm{kin}}+\eta_{\mathrm{ohm}}+\eta_{\mathrm{mass}}$$

The structure of the MEA plays a crucial role in determining water electrolysis performance. Key factors such as catalyst layer composition, ion exchange membrane properties, and PTLs structure significantly influence reaction kinetics, ionic conductivity, and mass transport. An optimized MEA design ensures efficient charge transfer, reduces overpotential, and enhances gas evolution dynamics. For instance, coating catalysts directly onto the IEM improves interfacial contact and minimizes resistance, leading to higher electrolysis efficiency. Additionally, tuning the porosity and hydrophilicity of the catalyst layer can further enhance mass transport and bubble detachment, ultimately boosting overall performance. Next, we will specifically introduce the recent advances in design hierarchical structures within membrane electrode assembly for PEM/AEM water electrolysis.

## 3 Design Mechanisms of MEAs with Hierarchical Structure

To move beyond empirical structural design, recent advances in MEA engineering have increasingly focused on elucidating and exploiting key physical and chemical mechanisms that dictate the integrated behavior of multi‐component MEAs. These mechanisms, uniquely applicable to MEAs rather than individual catalytic materials, govern interfacial charge transfer, mass transport matching, and proton/hydroxide conduction continuity across heterogeneous layers. Here, we summarize several representative strategies and mechanistic concepts.

### 3.1 Nested Interface Mechanism

The conformal coupling between the PTL/membrane and CL is critical for minimizing contact resistance and maintaining continuity of the triple‐phase boundary (TPB). Such intimate interfacial contact ensures efficient transfer of electrons, protons (or hydroxide ions), and reactant gases across the CL‐PTL/membrane interface, which is essential for sustaining high reaction rates, especially under industrial‐level current densities. Unlike traditional MEAs (with flat‐layered structure), nested architecture (e.g., interdigitated micro‐arrays or occlusal patterns) can significantly enhance interfacial coupling.^[^ ^44^ ^]^ These spatially engineered designs promote mechanical interlocking and increase the effective contact area, reducing interfacial voids and charge transfer bottlenecks.

Moreover, the hierarchical alignment of microstructures in these nested architectures reduces dead zones and preserves the vertical orientation of electron, ion, and gas transport pathways. This well‐defined directional transport minimizes tortuosity and enhances the uniformity of local reaction environments, thereby lowering activation losses. In addition, the presence of interpenetrating geometries facilitates rapid detachment and removal of gas bubbles, which is particularly beneficial under high current operation where mass transport limitations and bubble accumulation become more pronounced. Altogether, these structural and mechanistic advantages contribute to improved MEA performance, durability, and scalability in practical electrolyzer applications.

### 3.2 Mass Transport Matching Mechanism

In AEM/PEM water electrolysis systems, efficient mass transport is crucial for maintaining high reaction rates and achieving durable operation, especially under high current densities.^[^ ^45^, ^46^ ^]^ From a structural perspective, the architecture of MEA, including the catalyst structures, CL, ionomer distribution, membrane surface and PTL, directly governs the transport of water, ions, and gaseous products.

Specifically, in AEM electrolysis, the primary mass transport pathways include the migration of hydroxide ions, the diffusion of water from the feed compartment into the CL, and the removal of generated hydrogen and oxygen gases. For PEM electrolysis, similar challenges exist, but the transport of protons dominates ionic conduction, and the membrane typically has higher water uptake and conductivity. Regardless of the electrolyte type, the spatial management of these transport processes is inherently linked to the multiscale structure of the MEA.

First, structurally, an effective CL must simultaneously ensure sufficient ionic conductivity, reactant accessibility, and gas release.^[^ ^47^, ^48^ ^]^ In particular, micropores within the CL facilitate intimate contact between the catalyst and ionomer, enhancing ion conduction and catalyst utilization. Mesopores serve as intermediate channels for the diffusion of water and dissolved gases. Macropores, particularly when aligned vertically or laterally, facilitate gas escape, helping to minimize bubble accumulation and flooding. This hierarchical pore architecture is especially important under industrial current densities (>1 A cm^−2^), where transport limitations become more severe.

Second, the distribution of ionomer within the CL also plays a critical role.^[^ ^7^ ^]^ In homogeneous ionomer coverage can lead to uneven ionic conduction and catalyst isolation, whereas overloading ionomers may block pores and hinder mass transport. Therefore, a structurally optimized ionomer‐catalyst interface is key to enabling continuous water supply and efficient ionic pathways without sacrificing porosity.

Third, the interface between the CL and the membrane or PTL introduces additional structural considerations.^[^ ^49^ ^]^ At the membrane‐CL interface, a dense and well‐bonded contact is needed to minimize interfacial resistance and facilitate fast OH− or H⁺ transport. At the CL‐PTL interface, mechanical compression, pore alignment, and surface roughness collectively affect how efficiently gas can be removed and fresh water can be replenished.

In summary, mass transport in AEM/PEM electrolysis is governed not only by intrinsic material properties but also by the structural configuration of MEA components and interfaces. Rationally designed hierarchical porosity, well‐balanced ionomer distribution, and carefully engineered interfaces are essential to overcome mass transport limitations and enable high‐efficiency water electrolysis. Future MEA designs should adopt an integrated structural strategy that couples nanoscale catalyst engineering with mesoscale porosity control and macroscale interface optimization.

### 3.3 Ionic Pathway Engineering

Ionic conduction in MEAs is highly directional, which protons in PEMs or hydroxide ions in AEMs must traverse CL efficiently to reach the reaction sites, while avoiding short‐circuiting, ion leakage, or dehydration. This directional transfer is essential to sustaining high current densities with minimal losses. To address this, recent studies have employed vertically aligned ionomer domains or graded ionomer distributions to construct anisotropic ion‐conduction pathways.^[^ ^50^ ^]^ These designs not only enhance through‐plane conductivity but also suppress in‐plane ion diffusion that can lead to efficiency losses or local pH gradients.

Moreover, the integration of conductive gradients, either via spatial variation in ionomer content or by embedding functional additives, has shown promise in modulating the local ionic environment. In parallel, permittivity modulation through controlled micro/nano‐structure or heterogeneous interfaces enables redistribution of the internal electric field.^[^ ^51^ ^]^ This helps focus field lines and confine the active reaction zones to optimal regions within the MEA. This spatial confinement minimizes parasitic side reactions, reduces crossover effects, and enhances turnover frequency by ensuring that ions and electrons meet only where needed. Together, these strategies represent a shift from passive ion transport to actively guided conduction, enabling greater efficiency and selectivity in advanced MEA designs.

### 3.4 Multiphase Interfacial Modulation

The electrochemical reactions in MEA are complex processes, involving solid (catalyst/support), liquid (electrolyte/ionomer), and gaseous (O_2_/H_2_) components that must interact synergistically within a confined and dynamic microenvironment. The efficiency of electrochemical reactions relies heavily on the precise orchestration of these three phases at the solid‐liquid‐gas interface.^[^ ^39^ ^]^ Key strategies include maintaining nanometer‐thin hydration films to support ion transport while avoiding gas flooding, and tailoring surface wettability to selectively promote water infiltration or gas release, depending on local operating conditions.

For instance, advanced structural motifs such as amphiphilic coatings or Janus structures have been incorporated into the CLs.^[^ ^52^ ^]^ These materials possess distinct hydrophilic and hydrophobic regions, enabling dynamic modulation of local wettability in response to changes in current density, temperature, or reactant flux. Such adaptive wettability enhances multiphase interactions by improving reactant accessibility and TPB continuity. These design strategies help establish a finely balanced and responsive microenvironment that is critical for the sustained high‐performance operation of MEAs.

## 4 Types of Hierarchical Structure in MEA

The MEA is the core component of AEM/PEM water electrolyzers. To optimize performance, durability, and efficiency, the hierarchical structures are incorporated at different scales within the MEAs. These hierarchical structures are designed to enhance mass transport, ion conductivity, catalyst utilization, and durability during water electrolysis.^[^ ^53^, ^54^, ^55^, ^56^, ^57^ ^]^ Based on the component of MEAs, we will discuss the hierarchical structures from the structure of catalysts itself, CLs, IEMs surface, and PTLs in the next content.

### 4.1 Catalysts with Hierarchical Structure

Catalysts in the MEA are crucial for overcoming reaction energy barriers in electrochemical reactions (HER and OER), directly influencing the energy consumption of the electrolyzer. Noble metal‐based materials, such as Pt/C and IrO_2_, are commonly applied as cathode and anode catalysts, respectively, due to their high activity and long‐term durability. However, their scarcity and high‐cost limit large‐scale applications. Therefore, many studies have focused on reducing the loading of noble metals or finding inexpensive non‐noble metal catalysts as alternatives while maintaining high performance.^[^ ^58^, ^59^, ^60^, ^61^ ^]^ Amongst the various approaches, regulating catalyst structure is one of the commonly used and effective methods to improve catalytic performance.^[^ ^62^, ^63^ ^]^ Compared to conventional nanoparticle structures, hierarchical structures offer several distinct advantages due to their multi‐scale architecture, which can obvious improve active surface area, enhance catalyst utilization efficiency, accelerate charge transfer and increase mass transport.^[^ ^64^, ^65^, ^66^, ^67^, ^68^ ^]^

#### 4.1.1 Active Surface Area Enhancement

**Figure 4:**
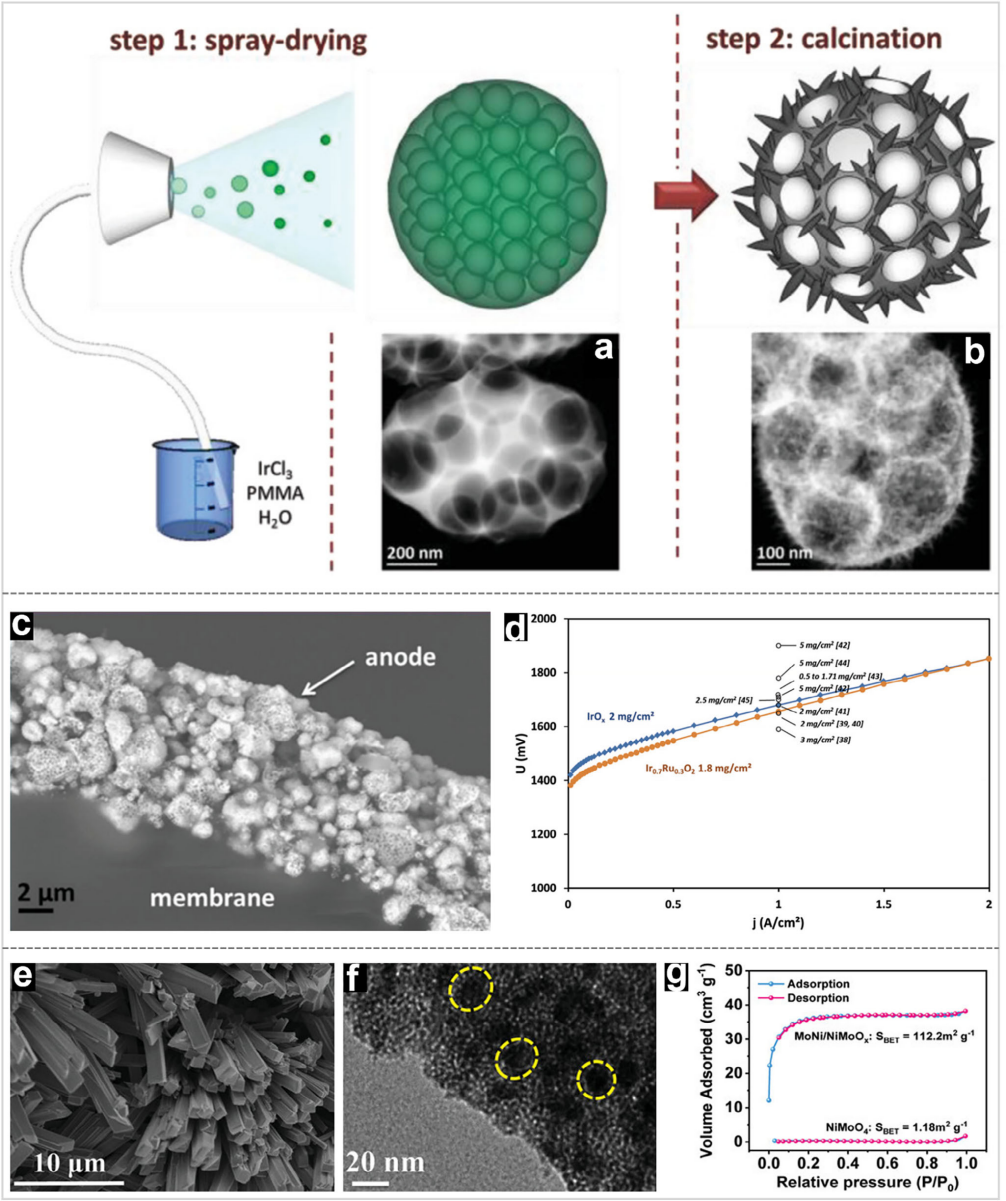
(a‐b) Schematic representation of the synthesis processes of the sample: a) after spray‐drying (step 1) and b) after calcination (step 2) at 450 °C. c) Post‐testing scanning electron microscope (SEM) images of a MEA and d) Polarization curves. e) SEM and f) transmission electron microscope (TEM) image of MoNi/NiMoOx. g) Nitrogen adsorption–desorption isotherms of catalysts. Reproduced with permission.^[^ ^79^, ^88^ ^]^ Copyright 2019 and 2023, Wiley‐VCH.

The first critical advantage of hierarchical structured catalysts is their significantly increased active surface area, which directly contributes to enhanced catalytic performance.^[^ ^69^, ^70^, ^71^, ^72^, ^73^ ^]^ The multi‐scale pore architecture, combining micropores, mesopores, and macropores, maximizes the exposure of active sites, thereby improving the overall efficiency of catalytic reactions. In conventional catalysts with limited porosity, many active sites remain buried within the bulk material, inaccessible to reactants. Hierarchical structures overcome this limitation by creating highly accessible surface areas, where reactants can easily interact with catalytic sites.^[^ ^74^, ^75^, ^76^ ^]^ In particular, micropores offer abundant active sites due to their large internal surface area, while mesopores and macropores facilitate the effective transport of reactants to these sites.^[^ ^77^ ^]^ This synergistic effect results in improved reaction rates. For example, Lei and co‐workers fabricated a hierarchical 3D NiFe‐alloy/NF catalyst through a simple and cost‐effective hydrothermal and followed by an electrodeposition method. Benefiting from the porous microstructure, high surface area, and exposed active sites, the corresponding AEM electrolyzer achieved a low cell voltage of 1.42 V at 10 mA cm^−2^ along with robust stability over 100 h.^[^ ^78^ ^]^ In another case shown in **Figure** **4**a,b, Peron and his collaborators reported hierarchically structured ultraporous iridium‐based materials based on an evaporation self‐assembly mechanism.^[^ ^79^ ^]^ They confirmed the rationally designed porous hierarchical structure optimizes the accessibility of reactants and products to the surface of the nanoparticles and maximizes catalyst activity, achieving the lower cell voltage of 1.65 V at 1.0 A cm^−2^ (Figure 4c,d). Similar functions based on hierarchical structures have also been demonstrated in previous reports.^[^ ^73^, ^80^ ^]^

Furthermore, the increased surface area enhances active site density, which is crucial for reactions occurring at high current densities.^[^ ^84^ ^]^ For example, catalysts with hierarchical structure exhibit higher current outputs at the same overpotential compared to their non‐porous counterparts.^[^ ^85^, ^86^, ^87^ ^]^ In addition, the hierarchical structure promotes uniform distribution of active materials, minimizing agglomeration and ensuring consistent catalytic activity over extended operation periods. For instance, Shi et al. designed a hierarchical crystalline/amorphous MoNi/NiMoO_x_ hydrogen evolution catalyst based on a hydrothermal and followed by an in‐situ thermal reduction strategy.^[^ ^88^ ^]^ The unique hierarchical structure of MoNi/NiMoO_x_ composites enables a highly active heterogeneous interface through the synergistic effect of different building units (Figure 4e‐g). When used as a cathode catalyst, the corresponding MEA achieved a high H_2_ production rate of 12.12 L h^−1^, 12.1 times that of commercial Ni foam and exhibited ultralong stability of 1600 h, verifying its great potential for industrial hydrogen production.

#### 4.1.2 Catalyst Utilization Enhancement

The second key advantage of hierarchically structured catalysts is their ability to improve catalyst utilization efficiency and reduce the cost of water electrolyzers while maintaining performance. Catalyst utilization refers to the proportion of active catalytic sites that are effectively involved in the reaction.^[^ ^89^, ^90^, ^91^ ^]^ In conventional catalysts, many active sites remain inaccessible due to poor material dispersion, particle agglomeration, or limited mass transport. Hierarchical structures can effectively address these issues through their multi‐level structure architecture. The optimized dispersion of active sites within hierarchical structures ensures that catalytic materials, especially noble metals like Pt or IrO_2_, are uniformly distributed across the support surface. This reduces agglomeration, which often leads to the deactivation of active sites in traditional catalyst designs.^[^ ^92^, ^93^, ^94^, ^95^, ^96^ ^]^ The combination of macropores for easy reactant access and meso/micropores for high surface area allows for maximum exposure of active sites, thereby enhancing utilization efficiency. For example, Shao and co‐workers fabricated hierarchically structured IrRu@WO_3_ nanoarray anode catalysts for PEM water electrolysis using a one‐step hydrothermal method.^[^ ^97^ ^]^ Benefiting from the hierarchical structure, the catalyst utilization efficiency is significantly improved, and the single cell can achieve a current density of 4.5 A cm^−2^ at 2.13 V with an Ir loading of 115 µg cm^−2^, as well as a long lifespan of 500 h at a current density of 0.5 A cm^−2^. Similarly, a core‐shell heterostructure combined with hierarchical structures also significantly improves catalyst utilization efficiency, resulting in decreased catalyst loading.^[^ ^98^ ^]^

#### 4.1.3 Charge Transfer Optimization

The multi‐level structure has also been demonstrated to promote faster electron and ion transport, leading to improved water electrolysis efficiency. In particular, hierarchical catalysts often feature interconnected networks of conductive materials, such as carbon frameworks, metal nanowires, or doped conductive oxides.^[^ ^99^, ^100^, ^101^ ^]^ These structures create continuous electron pathways that reduce electrical resistance and improve charge mobility. The presence of nanostructured conductive backbones (e.g., nanotubes, nanorods, Ni foam) within the hierarchical architecture allows for faster electron flow, facilitating rapid electron transfer to active sites during electrolytic processes.^[^ ^102^, ^103^, ^104^, ^105^ ^]^ This is particularly important at high‐current‐density operation, where electron transport limitations can significantly impact performance. For instance, Ghosh et al. reported a homogeneous bimetallic phosphide, Ni_2_P‐CuP_2_, on a Ni foam‐graphene–carbon nanotube (CNT) multi‐level structure using a facile electrochemical metallization method followed by phosphorization.^[^ ^106^ ^]^ Thanks to its interconnected network structure, this system achieved very low charge transfer resistance, along with high performance and excellent stability. Accordingly, Qin and his coworkers introduced an electric polyaniline nanofibrous network into the cathode catalyst structure using a spraying and in‐situ deposition method.^[^ ^107^ ^]^ When fabricated cathode catalysts were used in the MEA, it achieved excellent performance of 1.77 V at 2 A cm^−2^ and 70 °C with a low Pt loading of 0.07 mg cm^−2^, which is comparable to that of a conventional cathode with 14 times higher Pt loading (1 mg cm^−2^).

**Figure 5:**
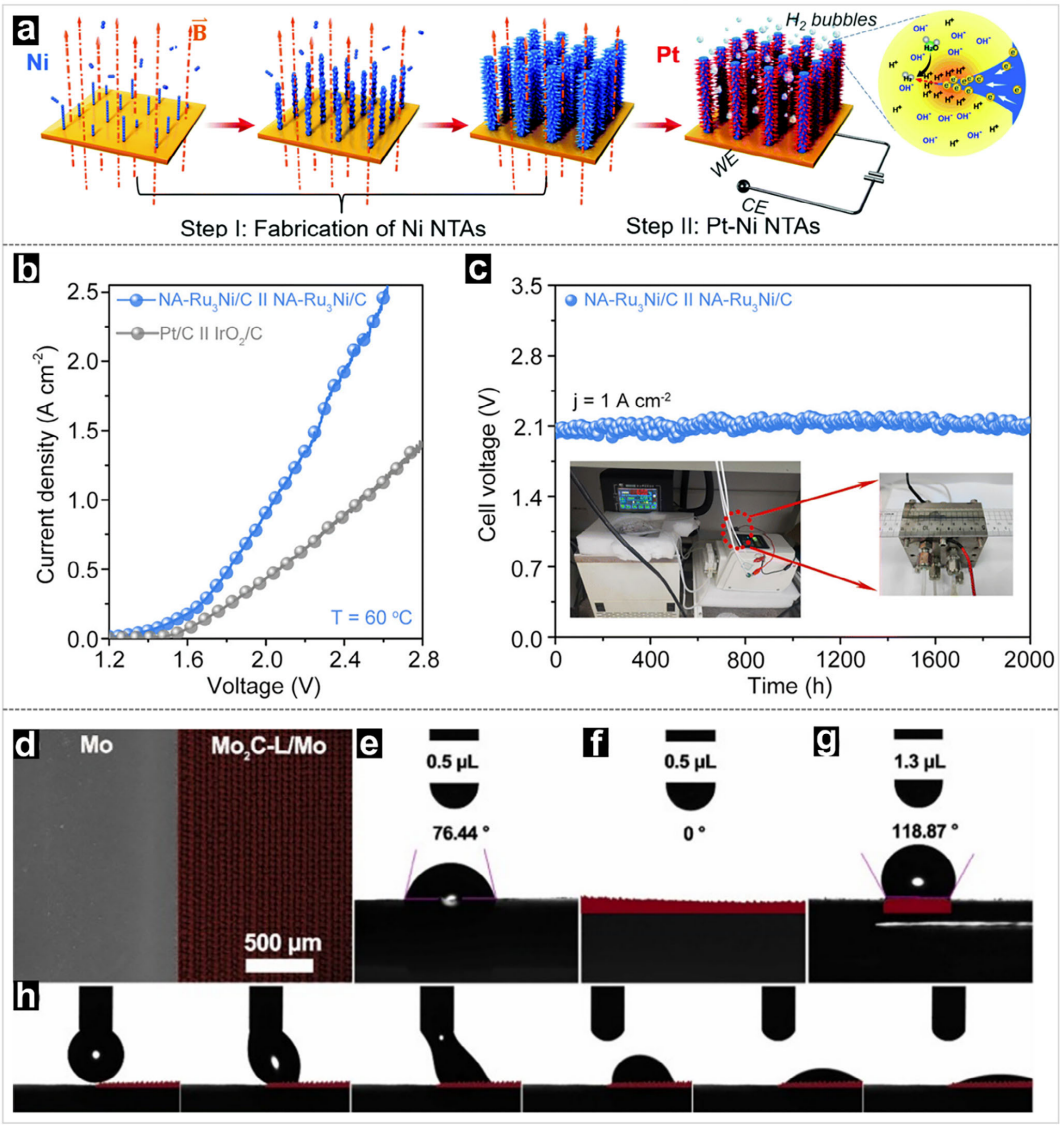
a) Schematic description of the fabrication process for the nano‐thorn structure through a magnetic field‐assisted growth process, the orange dotted lines are the magnetic field. Reproduced with permission.^[^ ^51^ ^]^ Copyright 2021, Royal Society of Chemistry. b) Polarization curve in an AEM electrolyser. c) Chronopotentiometry curve for AEM electrolysis operating at 1 A cm^−2^. Reproduced with permission.^[^ ^114^ ^]^ Copyright 2023, Royal Society of Chemistry. d) SEM image of the interface between Mo and Mo_2_C‐L/Mo micropillars. The surface contact angle measurements of the e) Mo, f) Mo_2_C‐L/Mo, and g) Mo|Mo_2_C‐L|Mo. h) The dynamic sessile drop contact angle measurement at the interface between Mo and Mo_2_C‐L. Reproduced with permission.^[^ ^116^ ^]^ Copyright 2022, Elsevier.

In addition to electron transport, hierarchical structures greatly enhance ion diffusion.^[^ ^108^, ^109^, ^110^, ^111^ ^]^ The combination of macropores (for bulk transport), mesopores (for intermediate diffusion), and micropores (for surface reactions) creates a multi‐level architecture that supports efficient ion movement throughout the CLs. This design minimizes ion concentration gradients and reduces the diffusion overpotential typically observed in dense CLs.^[^ ^112^ ^]^ For instance, Xu's group developed an anisotropic superstructure by in situ anchoring ultrafine ZnS nanoclusters on ordered macro‐microporous carbon skeleton (ZnS/SOM‐C).^[^ ^113^ ^]^ They investigated the mechanism of ordered macro‐micropores on OH− diffusion using the finite element method (FEM). They revealed that OH^−^ ions can hardly diffuse into the inner regions of the microporous ZnS/C, reducing the utilization efficiency of active sites and resulting in inefficient ion transport in the near‐surface region. In contrast, ZnS/SOM‐C features a 3D ordered macroporous superstructure and highly dispersed ZnS nanoclusters (average size of 9 nm), allowing OH^−^ ions to diffuse not only on the surface but also into the inner regions of ZnS/SOM‐C, which significantly improves faradaic ion transport and active site utilization. In another study, Yang et al. developed a unique nano‐thorn‐like Pt‐Ni nanowire electrode, which can exponentially increase the diffusion rate of H^+^ towards the electrode surface as compared with K^+^, which promotes faster reaction kinetics for the HER in an alkaline medium (**Figure** **5**a–c).^[^ ^51^, ^114^ ^]^ As a result, it achieved an extremely high HER performance towards real applications, with low overpotentials of 23 mV and 71 mV at current densities of 10 mA cm^−2^ and 200 mA cm^−2^, respectively.

Furthermore, the shortened ion transport pathways within hierarchical pores enable faster access of protons, hydroxide ions, and other charge carriers to the active sites.^[^ ^105^ ^]^ This is critical for enhancing the performance of AEM/PEM electrolyzers, where ion conductivity is a key factor. For example, Zhou and his collaborators fabricated a Pt‐anchored Mo_2_C micropillar electrode using a controllable laser ablation method.^[^ ^116^ ^]^ Due to more efficient H⁺ transport and shorter ion diffusion distances, the constructed MEA achieved a significant improvement in current density at the same cell voltage (Figure 5d–h). Moreover, the interplay between enhanced electron and ion transport leads to improved overall reaction kinetics. Faster charge transfer reduces the activation energy required for electrochemical reactions, decreases the overpotential, and increases the current density at a given voltage. This synergy also helps maintain performance stability during long‐term operation, as charge transport bottlenecks are minimized. For example, wood‐derived nanomaterials have been widely used as water electrolysis catalysts due to their hierarchically porous structure, which facilitates fast charge and ion transport.^[^ ^117^, ^118^, ^119^, ^120^, ^121^ ^]^

#### 4.1.4 Mass Transport Enhancement

**Figure 6:**
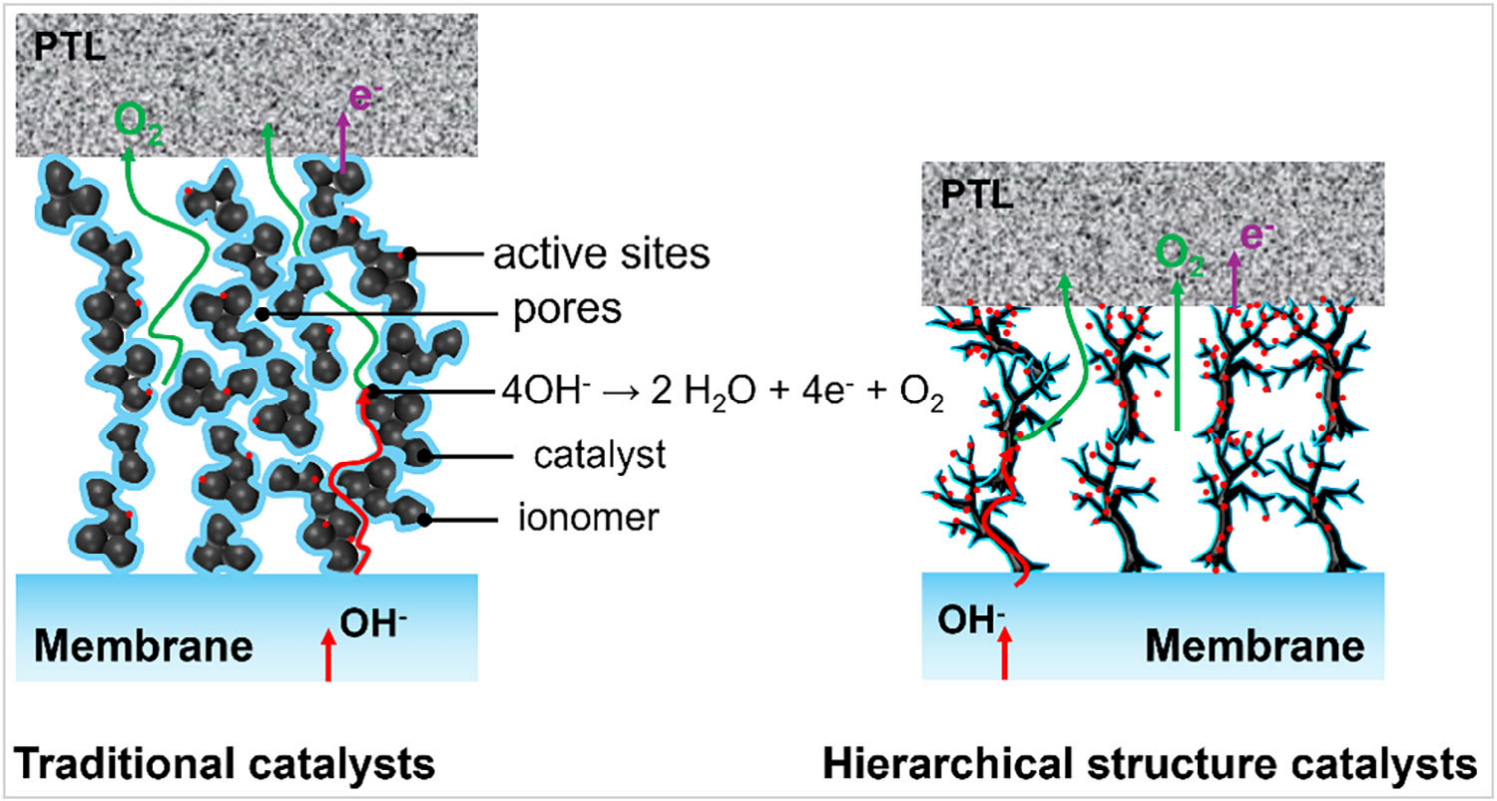
Comparison between the catalysts with hierarchical structure and traditional catalysts in mass–charge transport in MEA.

Another significant advantage of hierarchically structured catalysts is their ability to greatly enhance mass transport efficiency. This improvement is primarily attributed to the presence of a well‐organized network of interconnected structures. For example, the pore structures at different scales‐micropores (<2 nm), mesopores (2‐50 nm), and macropores (>50 nm), which collectively create efficient pathways for reactant diffusion and product removal.^[^ ^122^, ^123^, ^124^ ^]^ As shown in **Figure** **6**, in traditional catalysts with uniform or poorly defined porosity, the diffusion of reactants to active sites is often hindered, especially under high reaction rates or within thick catalyst layers.^[^ ^125^, ^126^, ^127^ ^]^ This leads to concentration polarization, where the reactant concentration near the active sites significantly decreases, thereby limiting catalytic performance. Hierarchical structures address this issue by providing short diffusion pathways through macropores, which act as highways for rapid mass transport, while mesopores and micropores offer high surface areas that promote active site exposure and facilitate intermediate molecule transport.^[^ ^128^ ^]^ Moreover, the interconnected pore network reduces tortuosity, facilitating faster and more uniform distribution of reactants throughout the catalyst matrix. This is particularly critical in water electrolyzers, where both ion and gas transport are essential for sustaining high current densities.

Numerous studies have shown that hierarchical catalysts can maintain superior performance even under conditions where conventional catalysts suffer from severe mass transport limitations.^[^ ^129^, ^130^ ^]^ Moreover, efficient product removal, such as the rapid desorption of O_2_ or H_2_ gas bubbles in water electrolysis, minimizes the blocking of active sites and reduces local pH fluctuations, further stabilizing reaction kinetics.^[^ ^131^, ^132^, ^133^ ^]^ This comprehensive enhancement in mass transport contributes to higher catalytic activity, improved efficiency, and longer operational stability. For instance, Liang et al. demonstrated that integrating conformal gas diffusion electrodes with graded wettability and porosity can significantly promote oxygen bubble release, reduce interfacial resistance, and suppress mass transport barriers at the CL‐PTL interface.^[^ ^134^ ^]^ Their work highlights the importance of wetting‐controlled interfacial continuity in maintaining long‐term MEA performance under dynamic operating conditions.

### 4.2 Multi‐Level Structured CLs

Apart from the catalyst itself, some studies have revealed that hierarchically structured designs for the CLs in the MEAs achieve significant improvements in catalytic activity, enhance mass transport, and optimize ion and electron conduction during the electrolysis process.^[^ ^135^, ^136^, ^137^, ^138^, ^139^ ^]^ In particular, a well‐designed CLs should balance proton, electron, reactant, and products transport. Hierarchically structured CLs that integrate multi‐level designs can achieve these targets, thereby improving electrolysis efficiency.

#### 4.2.1 Gradient Distributed Ionomer and Catalysts

In traditional CLs, the uniform distribution of ionomers and catalysts often leads to suboptimal trade‐offs between ion conduction and reactant/product transport.^[^ ^140^ ^]^ Excessive ionomer content can block active site exposure, while insufficient ionomer limits ion conductivity in the CLs. CLs with hierarchical structures, such as gradient composition designs, address this issue by spatially varying the ionomer content, catalyst loading, and porosity to create distinct functional zones within the CLs. This tailored structure ensures that each region performs optimally based on its proximity to the IEMs or PTLs.

**Figure 7:**
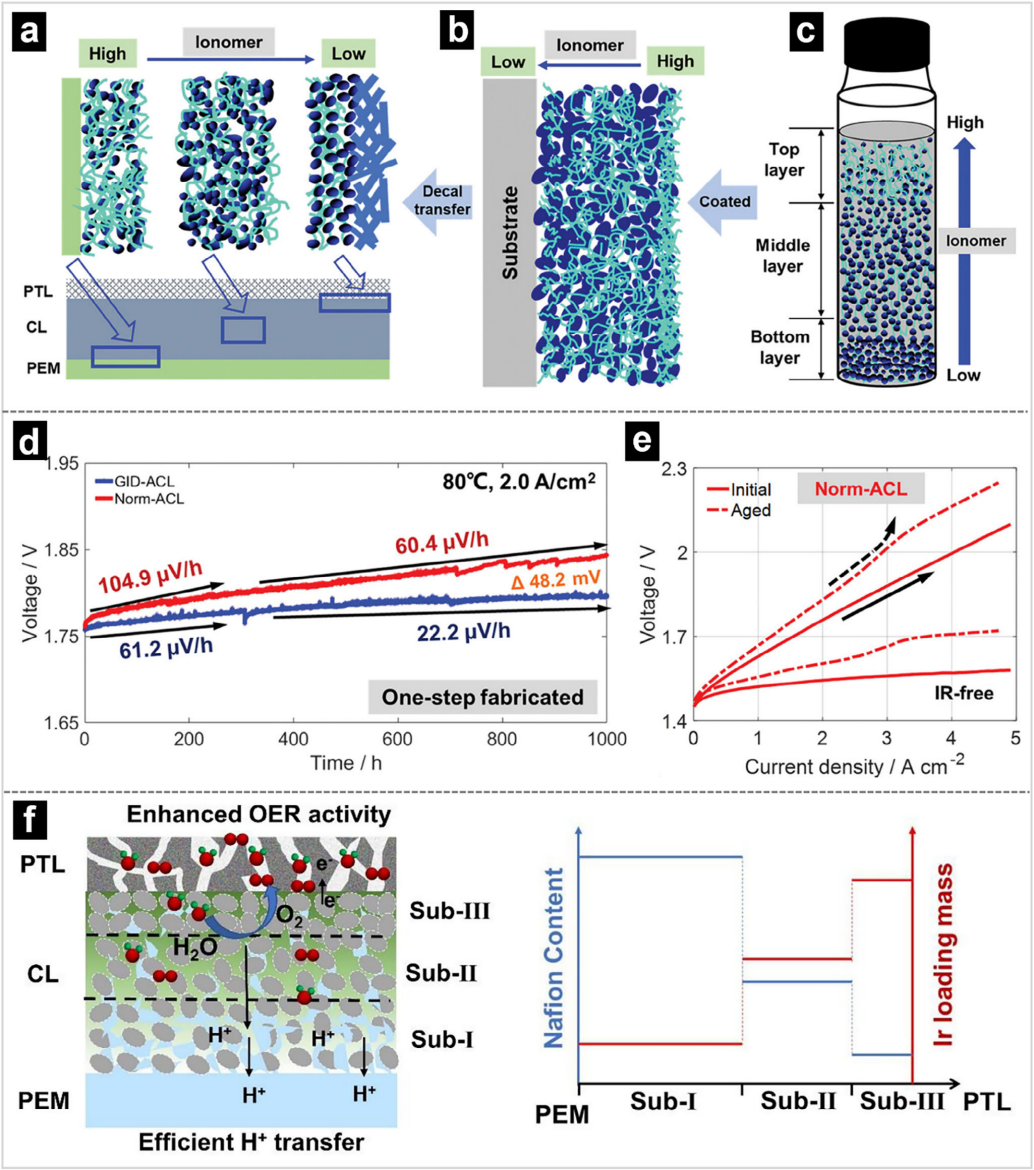
a) The designed anode CL (ACL) structure with enriched ionomer at ACL/PEM interface and reduced ionomer at ACL/PTL interface. b) The intermediate ACL on the decal transfer substrate. c) The illustrated ionomer/catalyst distribution of the catalyst ink for ACL. d) Durability test of Norm‐ACL and ACL at 2.0 A cm^−2^. e) Polarization curves of initial and aged Norm‐ACL. Reproduced with permission.^[^ ^143^ ^]^ Copyright 2024, Wiley‐VCH. f) The schemes illustrate a catalyst layer with a three‐sublayer structure. Reproduced with permission.^[^ ^145^ ^]^ Copyright 2024, Elsevier.

A common approach is to reduce the ionomer content near the PTL to facilitate reactant access, while increasing it near the membrane to promote efficient H^+^/OH^−^ conduction. This gradient minimizes gas/liquid transport resistance without compromising H^+^/OH^−^ conductivity.^[^ ^141^, ^142^ ^]^ For instance, a study by Zheng and coworkers demonstrates that reducing ionomer content at the anode CL/PTL interface while enriching ionomer content at the anode CL/proton exchange membrane (ACL/PEM) interface can significantly improve the lifespan of the MEA.^[^ ^143^ ^]^ As displayed in **Figure** **7**, the study reveals that ionomer content should be reduced at the interface between the PTL and ACL, as electron transfer and mass transport play a dominant role in overall performance. In contrast, proton transfer is the most critical factor at the interface between the PEM and ACL. As a result, the optimized MEA achieved a threefold reduction in the lifespan decay rate when operated at 2.0 A cm^−2^ and 80 °C.

Accordingly, varying the catalyst loading across the CLs can enhance catalyst utilization.^[^ ^144^ ^]^ A higher catalyst density near the membrane improves reaction kinetics, while a lower loading near the PTL reduces mass transport resistance. In this regard, Lv et al. designed a CL with a three‐sublayer structure by spraying catalyst inks of different concentrations, where each sublayer differs in ionomer content and IrO_2_/TiN_x_ ratio.^[^ ^145^ ^]^ They found that a high ionomer content in the sublayer reduces the utilization of active sites in the catalyst layer. However, due to improved kinetic performance and proton conductivity near the membrane interface, and thus significantly improves the mass transport. In contrast, a higher Ir loading near the PTL and lower Ir loading near the membrane interface can effectively improve cell performance, especially at high current densities. Therefore, the internal voltage loss of the optimized MEA is 21.5% lower than that of conventional single‐layer CL MEAs at 3 A cm^−2^.

**Figure 8:**
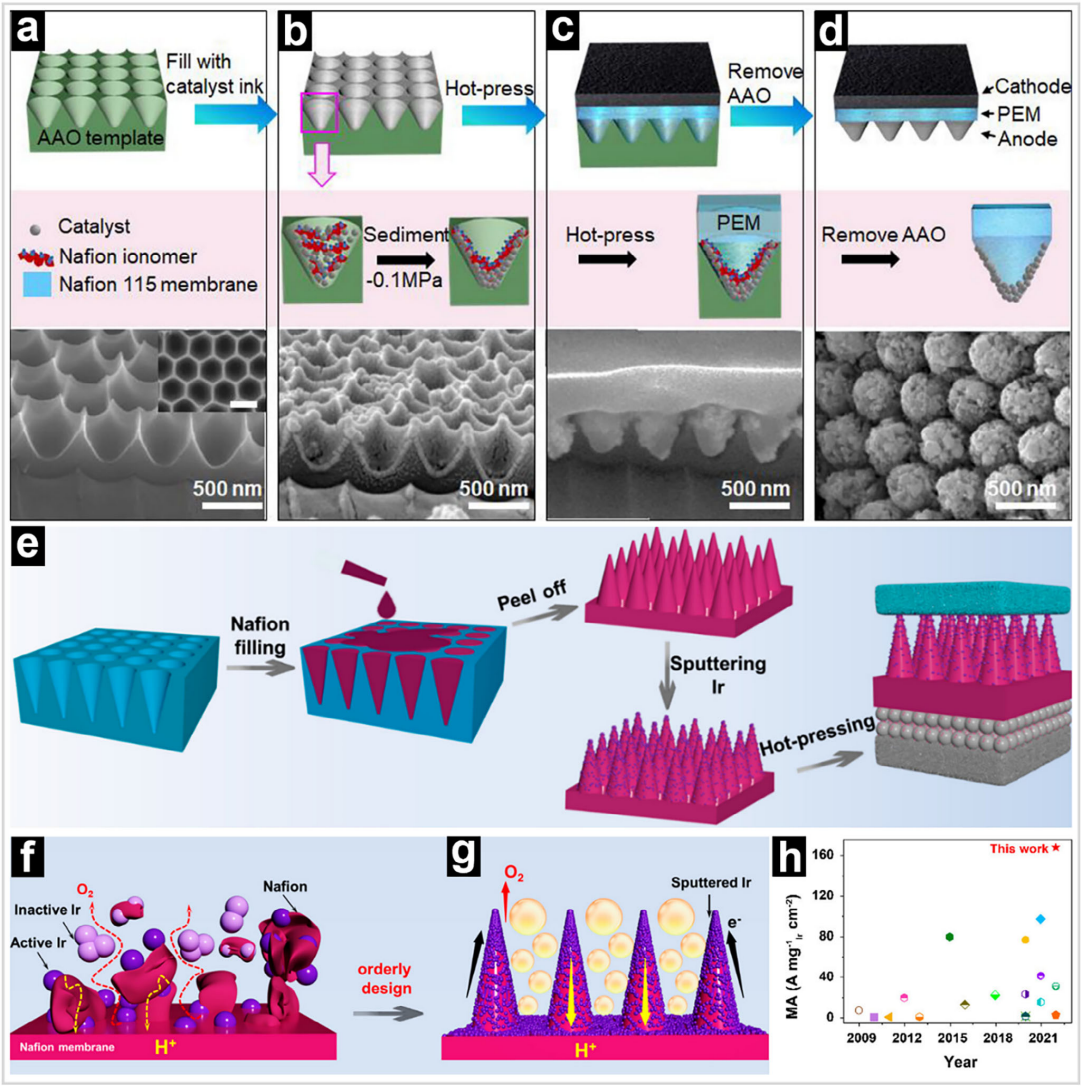
a) SEM image of AAO template. b) Cross‐sectional SEM image of the dried template filled with catalyst and ionomer. c) SEM image of the composite structure of the CL, PEM, and the template. d) SEM image of the tapered arrays after removing the template. Reproduced with permission.^[^ ^22^ ^]^ Copyright 2022, American Chemical Society. e) Step synthesis of ordered MEA. f,g) Schematic illustration of the improvements from a traditional MEA to an ordered MEA. h) Comparison of PEM water electrolysis mass activity of ordered MEA‐cone array with literature‐reported MEAs at 2.0 V. Reproduced with permission.^[^ ^42^ ^]^ Copyright 2023, American Chemical Society.

**Figure 9:**
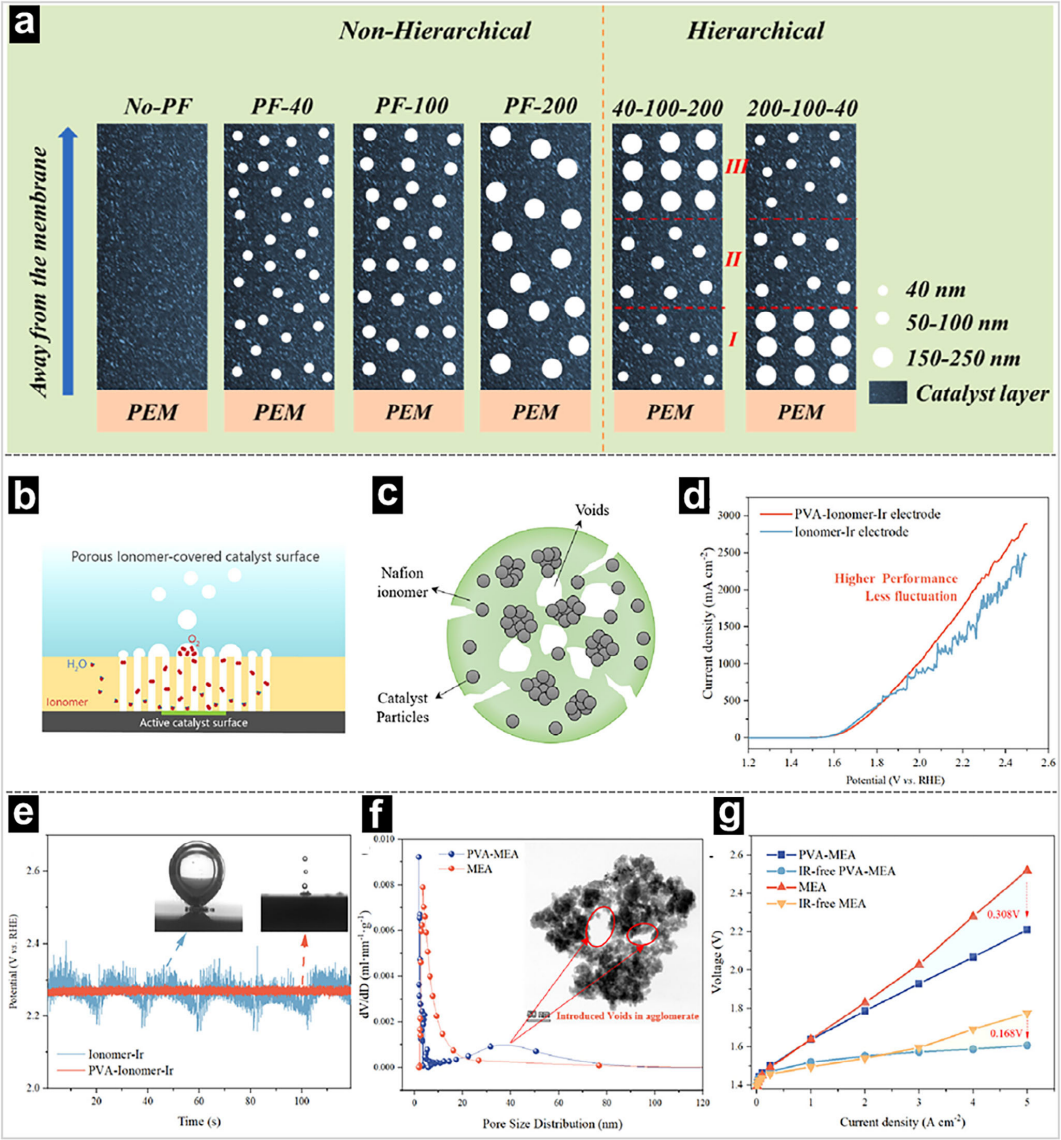
a) Schematic diagram of the ACLs with different pore‐forming structures. Reproducedwith permission.^[^ ^151^ ^]^ Copyright 2023, Elsevier. b) Scheme of the effect of the porous ionomer‐covered catalyst surface during the OER and bubble evolution. c) Scheme of the agglomerate structure optimized by the voids introduced. d) Polarization curves of the porous ionomer‐covered Ir (PVA‐Ionomer‐Ir) and Ionomer‐Ir electrodes. e) Changes in potential oscillation and bubble evolution behaviors during constant current operation. f) Pore distribution and TEM image of PVA‐modified porous agglomerate in the ACL. g) *I–V* and iR‐free *I–V* curves of MEAs. Reproduced with permission.^[^ ^145^ ^]^ Copyright 2024, American Chemical Society.

To avoid the stacked structure of the catalyst and ionomer, modifying the morphology of the ion exchange membrane through hot‐pressing or casting with templates is another effective method for fabricating CLs with gradient structures. A multiscale strategy combining ionomer tuning and pore gradient engineering was proposed by Zhou and coworkers,^[^ ^146^, ^147^ ^]^ where a graded ionomer distribution was shown to alleviate ionic conductivity bottlenecks and enhance reactant accessibility near the membrane interface. This approach further emphasizes that structure‐property relationships in MEAs are not merely geometric but intricately linked to chemical and electrostatic compatibility among all functional domains. Similarly, Yang and coworkers fabricated a gradient‐distributed CL on the surface of a cone‐shaped Nafion array using a heat transfer printing method (**Figure** **8**a–d).^[^ ^22^ ^]^ In our previous works, we designed the cone‐shaped Nafion array using a template method (**Figure** **9**e), which ensures a gradient distribution of the proton conductor from the membrane to the PTLs within the CLs.^[^ ^50^ ^]^ The ordered array structure not only remarkably improves the utilization efficiency of Ir catalysts but also facilitates gas and liquid transport during the electrolysis processes (Figure 8f,g). Therefore, it achieved a current density of 3.36 A cm^−2^ at 2.0 V with an Ir loading of 0.02 mg cm^−2^ (Figure 8h).

Although the use of cone‐shaped Nafion arrays or other membrane‐templating strategies offers an intriguing approach to achieving gradient CL architectures, there are several considerations that merit further clarification. First, modifying the ion exchange membrane morphology through hot‐pressing or templating may introduce mechanical stress or inhomogeneous thickness in the membrane, potentially affecting long‐term durability and ionic conductivity due to local water content and distribution.^[^ ^148^ ^]^ In particular, the gradient distribution of ionomer can enhance proton transport; however, excessive structuring may lead to localized hydration imbalance, especially under dynamic load conditions, potentially causing membrane dry‐out or flooding in confined regions. Therefore, although membrane patterning offers promising structural benefits, its influence on ion transport and mechanical stability must be carefully evaluated to ensure overall MEA performance and longevity.

#### 4.2.2 Gradient Distributed Pores

The pore size distribution in the CLs is another key factor in determining water electrolysis performance. Specifically, macropore‐dominant designs (100–200 nm or larger) facilitate rapid gas bubble removal and low tortuosity pathways, especially under high current density operations where water electrolysis is mass‐transport limited. For example, Faustini et al. demonstrated that hierarchically macroporous IrO_2_‐based electrodes with pore diameters >100 nm enabled efficient O_2_ evolution and minimized concentration polarization under 1.0 A cm^−2^ operation.^[^ ^79^ ^]^ Similarly, Liu et al. reported enhanced bubble detachment and gas permeability in MEAs using structured PTLs with large interconnected pores, improving performance at industrial current densities.^[^ ^149^ ^]^

Conversely, studies, for instance, Chatterjee et al.^[^ ^125^ ^]^ advocate for smaller pore sizes (5–10 nm), especially in densely packed CLs or in CLs close to the membrane, where enhanced water retention, ionomer connectivity, and higher proton/OH^−^ conductivity is critical. In such cases, excessive macroporosity may compromise active site accessibility or promote local dehydration. These discrepancies likely arise from differences in operating current densities, hydration conditions, CL thickness, and the relative position of pores within the CL. To provide a clearer picture, we have added **Table** **1** to compare the effect of different pore structures on PEMWE performance in the revised manuscript.

**Table 1:** Comparative summary of reported optimal pore sizes in CLs and their operational contexts.

Pore size	Pore region	Performance improvement	Reported benefits	Potential trade‐offs	Refs.
<5 nm	All CL	ECSA improves 8.7 times	Improves mass transfer and Ir utilization	Array stability	[42]
5–10 nm	All CL	Decreased ∼80 mV at 2 A cm^−2^	Decreases ohmic resistance and improves reaction kinetics	Larger mass transfer resistance	[125]
20–200 nm	All CL	Decreases ∼90 mV at 2 A cm^−2^	Improves catalyst utilization and electron transport	Structure collapse	[153]
40‐250 nm	Gradient CL	Decreases ∼234 mV at 3 A cm^−2^.	Improves mass transfer	Structure collapse	[151]
Average 50–70 nm	Gradient CL	Decreased∼234 mV at 3 A cm^−2^.	Improves kinetic properties and proton conductivity	Structure collapse	[145]
50‐100 nm	Gradient CL	Decreases ∼154 mV at 3 A cm^−2^.	Improves mass transfer	Structure collapse	[154]
100‐200 nm	All CL	Decreases 24 mV at 1 A cm^−2^	Prevents mass transport limitations and catalyst layer flooding	Structure collapse and CL detachment	[79]

Furthermore, recent studies have also demonstrated the “dual‐pore strategy” is a more effective method for designing high‐performance MEA, which integrates: macropores (>100 nm) to act as highways for gas and liquid movement, and mesopores (<50 nm) to ensure high interfacial area and proton/hydroxide transport.^[^ ^150^ ^]^ This synergistic approach appears particularly effective in mitigating the trade‐offs between mass transport resistance and electrochemical surface utilization. For example, Zhang and coworkers proposed an innovative configuration design of the ACL with three sub‐layers, featuring a hierarchical pore size distribution based on a chemical etching method (Figure 9a).^[^ ^151^, ^152^ ^]^ Further electrochemical characterizations suggested that the hierarchical structure of the ACL enhances the number of active sites, reaction kinetics, and mass transport, leading to superior electrochemical performance (2.0 V@3.0 A cm^−2^), which is 163 mV lower than that of the ACL without pore‐forming treatment (Figure 9b–g).

### 4.3 IEM surface structure

IEMs play a critical role in AEM/PEM water electrolysis processes by facilitating H^+^/OH^−^ conduction while preventing the crossover of gases such as H_2_ and O_2_. Recent research has focused on developing ion exchange membranes with surface micro‐nano structures to enhance the performance, efficiency, and durability of AEM/PEM electrolyzers.^[^ ^155^, ^156^, ^157^, ^158^ ^]^ Surface micro‐nano structures on IEMs aim to optimize the interface between the membrane and CLs or PTLs, improve H^+^/OH^−^ conductivity, enhance gas/liquid transfer, and resist mechanical degradation under harsh operating conditions. In this part, we will introduce the structure design of IEMs surface from gradient structure and dendritic structure, revealing corresponding functions in enhancing electrolysis performance.

#### 4.3.1 Gradient Structure

**Figure 10:**
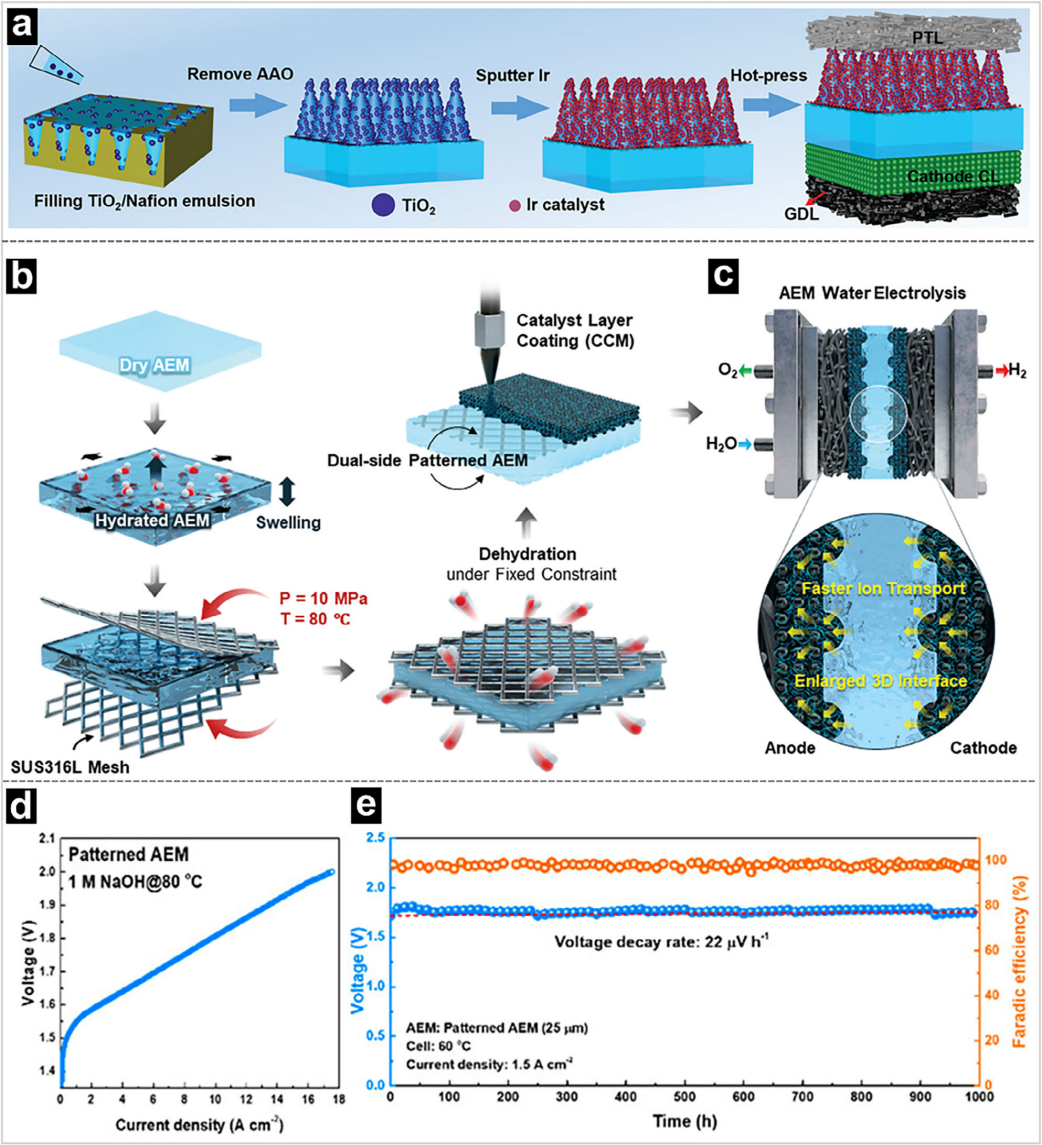
a) Preparation process and related characterization of hierarchically ordered MEA. Reproduced with permission.^[^ ^159^ ^]^ Copyright 2024, Wiley‐VCH. Schematics of b) fabrication process of dual‐side patterned AEM, and MEA with patterned AEM via direct catalyst deposition method, and c) Effects of dual‐side patterned AEM for water electrolysis. Reproduced with permission.^[^ ^160^ ^]^ Copyright 2025, Wiley‐VCH. d) The polarization of the patterned MEAs. e) Long‐term durability of MEAs. Reproduced with permission.^[^ ^145^ ^]^ Copyright 2024, American Chemical Society.

Surface gradient structures in IEMs for electrolyzers are designed to vary the microstructure across the membrane surface to optimize proton transfer and accelerate electrochemical reactions. For example, a cone‐shaped structure Nafion array has been designed to improve the proton transfer in the CL and improve noble metal Ir utilization efficiency.^[^ ^22^, ^50^ ^]^ Furthermore, we doped TiO_2_ nanoparticles into Nafion emulsion to fabricate a hybrid structured array, improving proton and mass transport in the CLs and achieving a current density of 2.48 A cm^−2^ at 2.0 V with an Ir catalyst loading of 14.4 µg cm^−2^ (**Figure** **10**a).^[^ ^159^ ^]^ Accordingly, Lee and his co‐workers proposed a facile method for preparing patterned membranes by casting a polymer solution onto commercially available monocrystalline silicon plates with pyramid‐shaped surface patterns.^[^ ^160^, ^161^ ^]^ The prepared membrane shows a 39% improvement in water permeability, significantly improving OH^−^ transfer (Figure 10b,c). As shown in Figure 10d,e, the patterned AEM achieves an unprecedented current density of 17.5 A cm^−2^ at 2.0 V using the catalyst‐coated membrane method. Moreover, the patterned AEM‐based MEA can operate at 1.5 A cm^−2^ and 60 °C for 1000 h with a relatively low voltage decay rate of 22 µV h^−1^.

Another primary strategy for creating surface gradients in IEMs is varying degrees of sulfonation. This increases the ions‐exchange capacity near the IEMs surface, enhancing H^+^/OH^−^ conductivity on the cathode side, where high proton flux is required, while maintaining a stable and robust bulk structure.^[^ ^162^, ^163^ ^]^ This gradient structure is crucial for maintaining long‐term stability and high efficiency during the electrolysis process. For example, polymer blending with surface gradient, in this approach, different polymer materials are used to create a gradient in the concentration of ion‐exchange groups on the surface of IEMs. This method combines high proton conductivity with improved mechanical properties by controlling the distribution of sulfonated and unsulfonated regions along the surface. However, in this review, we mainly focus on the microstructure of the IEMs surface, there are numerous reports on the regulation of IEMs structures at the molecular level.^[^ ^106^, ^164^, ^165^ ^]^ Plasma treatment is another technique used to modify the surface microstructure of IEMs, effectively improving catalyst utilization efficiency, charge and mass transport.^[^ ^166^ ^]^ By applying different plasma treatments (e.g., O_2_/N_2_ plasma), the surface characteristics can be tailored to control water retention and enhance the membrane's resistance to swelling and degradation under electrolysis conditions.^[^ ^167^ ^]^ For example, Kus and his collaborators fabricated a fiber‐like structure on the surface of the IEM using a dry sputter‐etching method with the help of a cerium oxide layer.^[^ ^168^ ^]^ The formed multi‐level structure significantly enhances the interface between the IEMs and CLs, improving charge transfer and achieving a current density of 4.0 A cm^−2^ at 2.0 V with a total precious metal loading of 0.22 mg cm^−2^.

#### 4.3.2 Dendritic Structure

**Figure 11:**
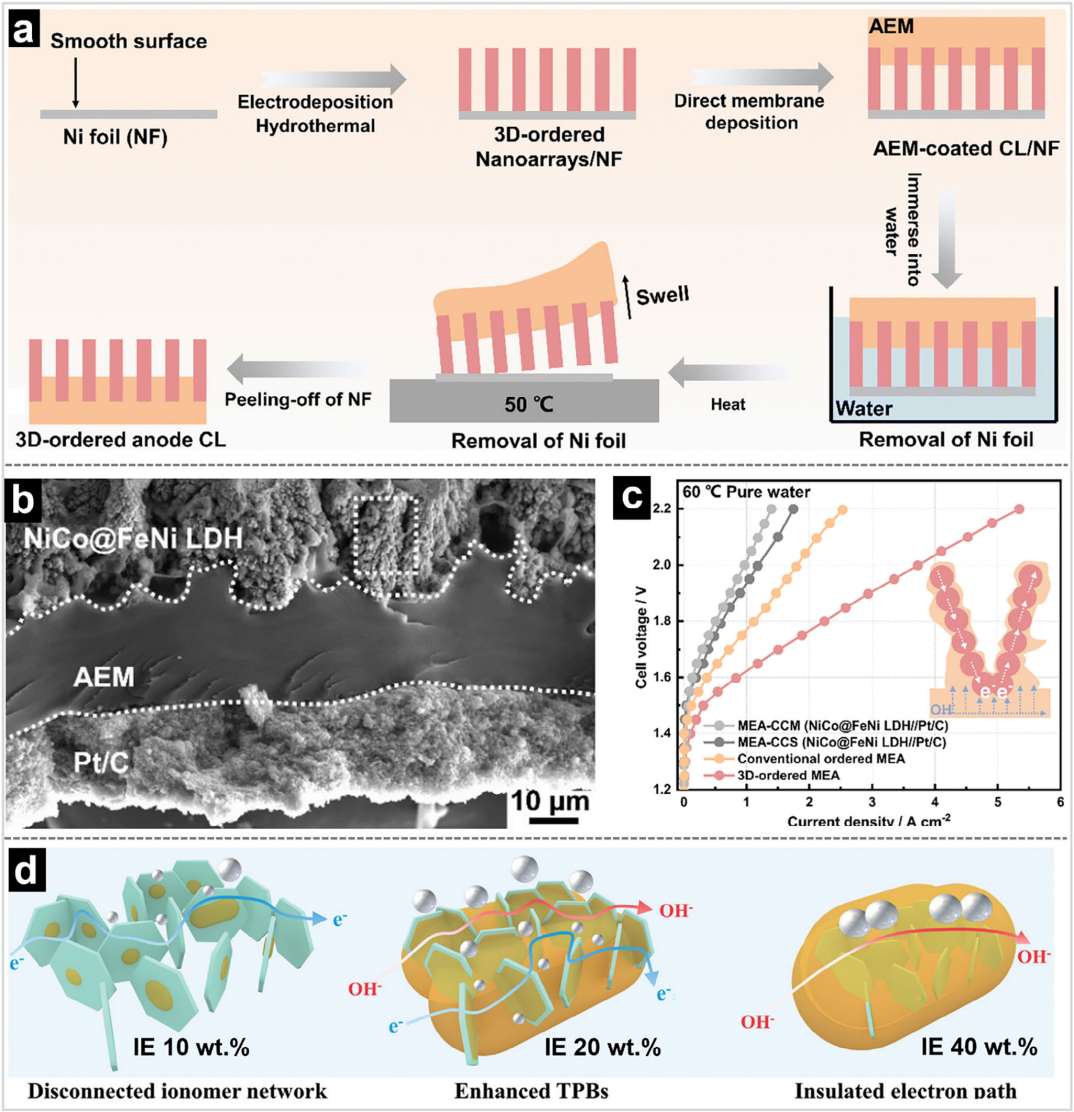
a) Schematic diagram for the novel swell‐assisted transfer method. b) Cross‐sectional SEM images of 3D‐ordered MEA based on NiCo@FeNi LDH porous foams as the anode CL. c) The performance of different MEAs. d) Schematic illustration of CL structures as a function of ionomer contents. Reproduced with permission.^[^ ^162^ ^]^ Copyright 2022, Wiley‐VCH. Reproduced with permission.^[^ ^171^ ^]^ Copyright 2024, Royal Society of Chemistry. Reproduced with permission.^[^ ^172^ ^]^ Copyright 2024, American Chemical Society.

The IEM with surface dendritic structure is another strategy to improve the catalysts utilization efficiency and the CL/IEM interface. As displayed in **Figure** **11**, Wang's group developed a series of all‐in‐one MEAs for highly efficient AEM water electrolysis by filling AEM emulsion into catalyst arrays.^[^ ^169^, ^170^, ^171^ ^]^ Benefiting from the porous structure of the catalyst array, the formed AEM surface exhibits a dendritic structure, which not only remarkably increases the reaction interface with the CLs but also forms a highway for OH− transfer. Using the prepared MEA in AEM water electrolyzers, it achieves a current density of 3.61 A cm^−2^ at 2.0 V under pure‐water‐fed conditions and stable operation for 700 h at a current density of 1.0 A cm^−2^ at approximately 1.7 V. These works provide a universal approach for constructing next‐generation MEAs for water electrolyzers.

### 4.4 Porous Transport Layer

PTLs are critical components in AEM/PEM water electrolyzers, functioning as current collectors and facilitating the efficient transport of reactants (e.g., water) and products (e.g., hydrogen/oxygen) across the electrode‐membrane interface. The performance of AEM/PEM electrolyzers heavily relies on the characteristics of the PTLs, and recent studies have focused on developing hierarchically structured PTLs to improve electrolysis efficiency, water management, and the overall durability of the electrolyzer.^[^ ^172^, ^173^, ^174^, ^175^ ^]^ In particular, hierarchically structured PTL designs aim to optimize gas and liquid transport, enhance electrical conductivity, and improve interactions with the CLs. For example, the gradient in pore size across the PTL helps facilitate the effective diffusion of gas products, ensuring that reactants (e.g., water vapor) can easily reach the catalyst sites while products (e.g., H_2_/O_2_) can quickly release and inhibit blockages or flooding.^[^ ^176^ ^]^ The gradient in hydrophobicity or hydrophilicity along the PTL helps control water distribution within the MEAs. Hydrophobicity near the CLs aids in removing excess water produced during the electrolysis reaction, while hydrophilicity near the membrane helps retain sufficient water for proton conductivity.^[^ ^177^ ^]^ Furthermore, the gradient in electrical conductivity across the PTL can improve current collection and reduce resistive losses, contributing to the overall efficiency of the PEM electrolyzer.^[^ ^178^ ^]^

#### 4.4.1 Microporous Layers

One of the most common approaches for optimizing the structure of PTLs is to vary the pore size along the thickness of the PTL.^[^ ^179^, ^180^, ^181^, ^182^, ^183^ ^]^ Near the CLs, smaller pores can provide higher capillary forces, aiding water retention, improving contact with the catalyst, and facilitating faster removal of gas bubbles, reducing the risk of local flooding. On the other hand, the larger pores are used to facilitate the transport of gases and reduce the likelihood of gas blocking. Therefore, this gradient structure helps balance gas diffusion and water management, leading to improved electrolysis efficiency, especially at high current densities.

**Figure 12:**
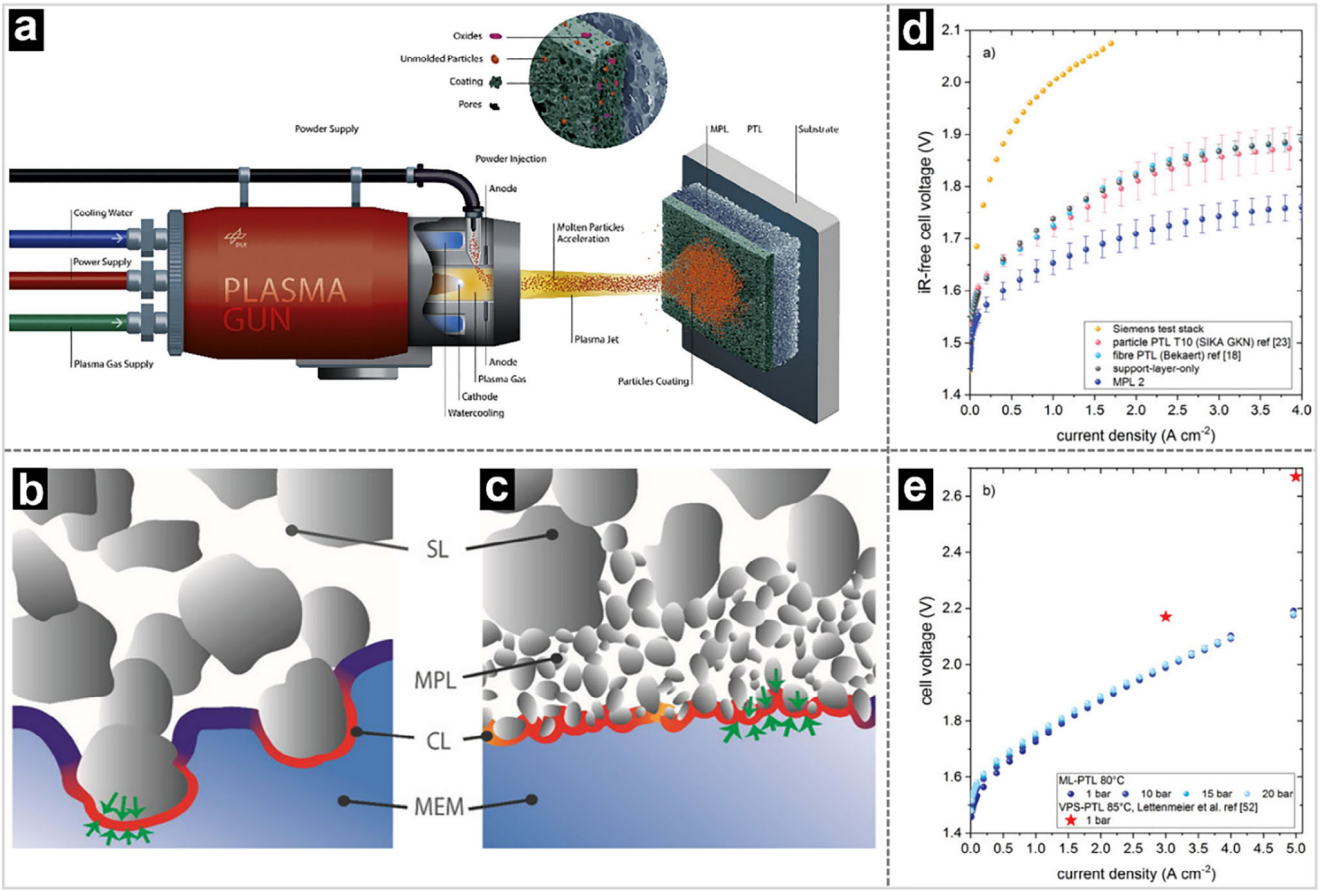
a) Schematic illustration of the coating of NiMPL on PTL. b,c) Local microscopic compression of CL by surface particles. Reproduced with permission.^[^ ^178^ ^]^ Copyright 2021, Elsevier. d) IR‐free polarization curves at 50 °C and 25 bar balanced gas pressure of water electrolysis test stack. e) Polarization curves of the laboratory cell with MPL 2. Reproduced with permission.^[^ ^179^ ^]^ Copyright 2020, Wiley‐VCH.

As presented in **Figure** **12**a, Friedrich and his coworkers loaded Ni‐based microporous layers (NiMPL) onto the surface of the PTL (NiMPL‐PTL) using air‐plasma spraying for pure‐fed AEM water electrolysis.^[^ ^184^ ^]^ The low tortuosity of this NiMPL‐PTL reduced the capillary pressure and bubble point, leading to lower transport polarization. Furthermore, the NiMPL‐PTL decreased the interfacial contact resistance by increasing the contact area between the PTL and CL. As a result, it achieved measurable performance improvements in the AEMWE operated in pure water: a 290 mV lower voltage at 0.5 A cm^−2^ compared to similar cells without the NiMPL‐PTL. Accordingly, Büchi et al. investigated the development of hierarchically structured PTLs for PEM water electrolyzers to enhance performance and reduce costs. They introduced multilayer PTLs with microporous layers made from titanium powders, designed to improve catalyst layer utilization, reduce mass transport losses, and enable the use of thinner membranes (Figure 12b,c).^[^ ^185^, ^186^ ^]^ As a result, significant improvements were achieved, including up to 2.8 times higher performance compared to conventional PTLs due to enhanced interfacial contact, an approximate 60 mV reduction at a current density of 2 A cm^−2^, and reduced surface roughness, which minimizes membrane deformation and enables the use of thin membranes (∼20 µm) (Figure 12d,e).

In addition, the gradient pore structure has also been controlled by the diameter of the fiber in the Ti felt.^[^ ^187^ ^]^ In short, the variation in pore sizes contributes to minimizing both ohmic losses (by maintaining effective ionic conduction paths) and concentration polarization losses (by enhancing mass transport). The gradient structure helps maintain optimal contact resistance with the CLs due to tighter pore networks, while providing better gas permeability through larger pores and minimizing concentration gradients during electrolysis at high current densities. Moreover, the gradual transition of pore sizes improves the PTL's mechanical integrity. Smaller pores near the membrane‐catalyst interface distribute mechanical stress more uniformly, reducing the risk of delamination or membrane damage. Larger pores provide structural flexibility, allowing the PTLs to accommodate thermal and hydration‐induced expansion without cracking. Similar conclusions have been demonstrated by other researchers.^[^ ^188^ ^]^

#### 4.4.2 Layer‐by‐Layer

In comparison with these random pore structures, ordered structures can further facilitate rapid gas bubble removal, ensure uniform reactant distribution, reduce electrical resistance, and improve overall water electrolysis efficiency, particularly at high current densities.^[^ ^189^, ^190^, ^191^, ^192^ ^]^ For example, different extrusion parameters can be applied across the PTL to vary pore sizes at each layer, achieving a gradual transition from small pores near the CLs to larger pores at the gas channel side. In particular, layer‐by‐layer control is an advanced fabrication strategy employed in the production of gradient PTLs for AEM/PEM electrolyzers. This technique leverages precise deposition methods to fabricate multi‐layered structures with tailored porosity, allowing for the gradual transition of pore size across the thickness of the PTL, which is especially effective for optimizing mass transport, water management, and electrochemical performance.

**Figure 13:**
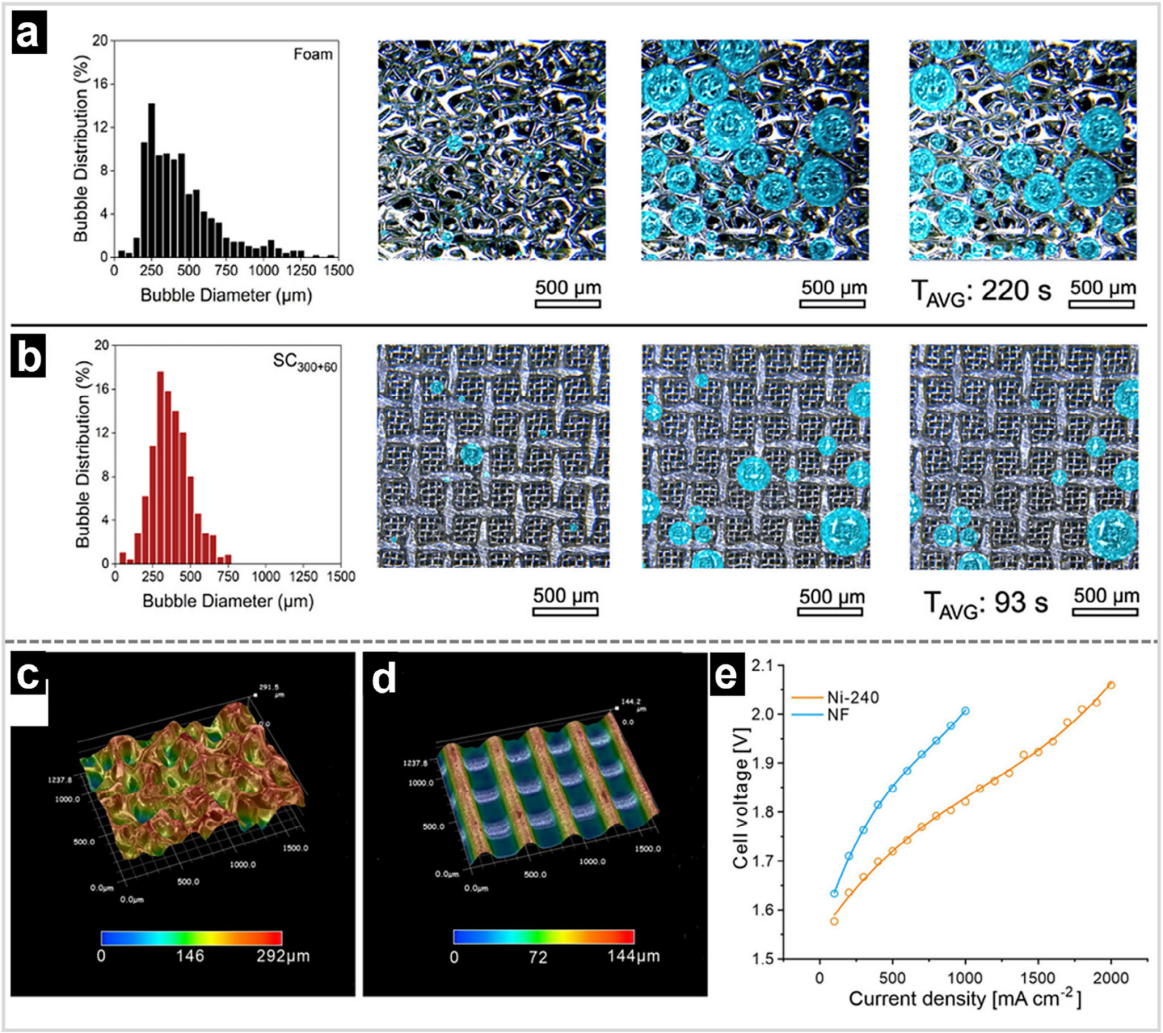
Bubble detachment distribution and camera screenshots from the different substrates at 8.26 mA cm^−2^ a) nickel foam, b) SC300+60, c) SC300, and d) SC60, where *T*_AVG_ is the average time for the bubble to detach from the substrates. Reproduced with permission.^[^ ^194^ ^]^ Copyright 2024, American Chemical Society. Optical microscope images of e) NF and f) Ni‐240. g) Performance comparison curves between NF and Ni‐240 in pure water at 80 °C. Reproduced with permission.^[^ ^195^ ^]^ Copyright 2023, Wiley‐VCH.

For example, Sun's group used a one‐step overlaying strategy to create a double‐layered nickel mesh substrate for enhancing the performance of the OER in AEM water electrolysis.^[^ ^193^ ^]^ As displayed in **Figure** **13**a–d, their study indicated that the introduced nickel mesh significantly improves mass transfer by creating open channels for the rapid transport of water and gases, which reduces concentration polarization and enhances the accessibility of active sites, especially under high current densities. Moreover, by facilitating better gas‐liquid separation and minimizing bubble accumulation on the electrode surface, the nickel mesh substrate reduces both ohmic and concentration polarization losses, leading to improved electrolysis performance. Thereby, a high current density of 5.01 A cm^−2^ at 2.0 V and 80 °C was achieved, surpassing most reported values. Similarly, Huang et al. and Zhuang et al. designed a PTL with hierarchical grid gradients for AEM water electrolysis using 3D printing techniques (Figure 13e,f).^[^ ^194^ ^]^ By optimizing the microstructure of the PTLs, the hierarchical grid gradient promotes efficient gas diffusion and reduces the likelihood of bubble accumulation on the electrode surface, thereby improving the overall electrolysis efficiency. Based on the fabricated hierarchically structured PTL, the corresponding MEA achieved a current density of 2 A cm^−2^ at a cell voltage of 1.95 V and 80 °C, one of the highest single‐cell performances in pure‐water‐fed AEM water electrolysis (Figure 13g).

#### 4.4.3 Surface Hydrophobicity

In AEM/PEM electrolyzers, effective water transport is critical because water serves both as a reactant and as a medium for H^+^/OH^−^ conduction.^[^ ^195^ ^]^ A hydrophobicity gradient helps maintain an optimal balance between water retention near the membrane (for hydration and H^+^/OH^−^ conduction) and efficient gas removal near the flow channels, minimizing mass transport losses. By introducing a gradient in surface properties, such as hydrophobicity, the PTLs can be tailored to improve water management and gas transport. Hydrophobic materials (e.g., polytetrafluoroethylene, PTFE) can be applied near the anode to prevent water accumulation, while hydrophilic materials can be used near the cathode/anode to promote water retention, which is necessary for H^+^/OH^−^ conduction in PEM/AEM electrolyzers. For example, Pan and his colleagues demonstrated that partially hydrophobic PTLs can achieve superior oxygen transport by promoting efficient bubble removal while maintaining adequate water supply for electrolysis reactions.^[^ ^196^ ^]^ Furthermore, Wilke et al. designed a PTLs with hierarchical wettability by loading a microporous layer.^[^ ^197^ ^]^ Similarly, Long et al. reported a dual‐path proton conduction mechanism enabled by nano‐confined ionomer channels embedded within a porous catalyst matrix, effectively enhancing both through‐plane proton mobility and in‐plane hydration stability.^[^ ^198^ ^]^ This approach represents a shift from isotropic ionomer networks to anisotropically guided ionic pathways, tailored for MEAs operating at industrially relevant current densities.″

In another case, Tang and his coworkers used laser micropatterning to create precise pore channels with controlled geometries in the PTLs to optimize wettability.^[^ ^199^ ^]^ Their study indicated that titanium felt inherently exhibits hydrophobicity; however, after laser processing, the formation of TiO_2_ on the surface surrounding the perforations enhances the hydrophilicity of PTL. In particular, the perforations are a micropatterned pore channel structure with numerous nanosized pores on the hole walls, which are irregular pores shaped due to the stacking of titanium fibers. The synergistic effect between the pore channels and the nanopores induces capillary action, resulting in strong hydrophilicity within the perforations, and thus improving mass transport. Another approach involves creating a gradient structure in material composition across the PTL. For example, PTLs may be fabricated with a combination of carbon‐based materials (e.g., carbon paper, carbon cloth) and conductive polymers to ensure high electrical conductivity and mechanical stability while optimizing gas and water transport properties.^[^ ^200^, ^201^ ^]^

Based on the above discussions, to offer a clearer view of the structure‐activity correlations in hierarchically engineered MEAs, we summarized key design strategies, their functional objectives, and corresponding performance outcomes in **Table** **2**. This table enables a comparative understanding of how specific micro‐/nano‐architectures contribute to performance enhancement, guiding rational MEA optimization.

**Table 2:** The effect of micro/nanostructure in MEA on performance.

Structural strategy	Key structural feature	Performance enhancement	Proposed mechanism	Potential trade‐offs	Refs.
Ordered micropillar arrays	Cone array and gradient CL	Voltage decreases ∼100 mV at 1 A cm^−2^	Enhances proton conductivity and mass transfer	Array stability	[159]
Pore gradient	Double‐layered nickel mesh PTL	Increases ∼2.3 times current density at 2 V	Accelerates gas bubble release	CL stability	[193]
Pore gradient	Hierarchically structured PTLs	Voltage decreases ∼100 mV at 7 A cm^−2^ at low Ir loading (0.4 mg cm^−2^)	Increases CL/PTL interface	Water/gas transfer limitation	[186]
Interdigitated Nested Interfaces	Interlocking between CL and membrane	Increases ∼3.4 times current density at 2 V, decay rate decreases 18.7 times at 1 A cm^−2^	Increases CL/membrane interface and mass transfer	Fabrication complex	[170]
Hierarchically structured catalysts	Hierarchical microporous structure	Voltage decreases 244 mV at 0.8 A cm^−2^	Facilitates gas/liquid transfer	Fabrication complex	[202]
Graded Ionomer Distribution	Decreases ionomer at CL/PTL and increases at CL/PEM interface	Decay rate decreases 3 times at 3 A cm^−2^	Provides ordered channels for charge and gas/liquid transport	Ionomer structure collapse	[143]
Hydrophobic‐Hydrophilic Janus	Amphiphilic gradient to control gas/liquid domains	Increases 1.5 times current density	Tuned multiphase interface, efficient gas escape	Fabrication complex	[198]

## 5 Summary and Outlooks

The MEA is a crucial component in AEM/PEM water electrolyzers, which directly determines the water electrolyzers performance and costs. One effective way to enhance MEA performance is by utilizing hierarchical structures within the components of MEAs, including the catalysts, CLs, IEMs surface, and PTLs. This review summarizes the recent progress in designing hierarchical structures of catalysts, CLs, IEMs, and PTLs in the MEAs to improve the corresponding water electrolyzers efficiency and lifespan. However, the integration of hierarchical structures within MEAs for water electrolyzers is still an evolving area of research, with promising directions for improvement.

### 5.1 Fabrication of Hierarchical Structure Components in MEAs

Hierarchical structures, characterized by multiple levels of organized porosity, have been shown to improve gas and liquid transport, reduce bubble accumulation, and enhance electrochemical reactions at the electrode interfaces. These structures, often achieved through advanced fabrication techniques such as template method, laser cutting, and material gradients, offer significant advantages, including accelerating gas/liquid transport, promote charge transfer, exposing catalytic active sites, increasing the lifespan of MEAs. However, the fabrication and microstructure regulation of hierarchical structure components in the MEAs is still a challenge due to the complex structure. Therefore, the development of more sophisticated manufacturing methods, such as 3D printing and laser micromachining, will enable more precise control over the microstructure of each MEA component, allowing for greater optimization of transport properties and increasing the electrochemical active sites, and then greatly improving the electrolysis efficiency as well as reducing the cost.

### 5.2 Structure Stability and Interface Regulation in MEAs

In practical applications, the long‐term operational stability of MEAs becomes increasingly important, often outweighing the benefits offered by sophisticated microstructure designs alone. Although hierarchical structures enhance catalytic performance and mass transport, they also introduce new structural vulnerabilities. For example, highly porous catalyst layers or ionomer gradients may suffer from mechanical collapse, catalyst detachment, or interfacial delamination under harsh electrolysis conditions such as high current density, elevated temperature, and fluctuating load.^[^ ^197^, ^203^ ^]^ Moreover, the interface mismatch between differently structured layers (e.g., CLs and IEMs) can compromise mechanical integrity and lead to increased voltage decay over time.^[^ ^204^ ^]^

To mitigate these issues, several stabilization strategies have been explored. Among them, an all‐in‐one design approach is necessary for boosting durable MEA performance. Recent studies have demonstrated several promising approaches toward this goal. For instance, occlusal‐interlocking structures, where the micro‐patterned CL or membrane conforms to the topography of the PTL, can significantly reduce contact resistance and mechanical delamination under high current operation.^[^ ^205^ ^]^ Likewise, gradient ionomer distributions within the CL, optimized through either solvent‐casting techniques, can balance proton conductivity and gas permeability, thereby improving local reaction environments.^[^ ^145^, ^202^ ^]^ Another representative method involves in situ co‐fabrication, where the CL and IEM are co‐cast or chemically bonded during the fabrication process, resulting in an intrinsically fused interface.^[^ ^206^, ^207^ ^]^ This method helps eliminate abrupt phase boundaries and enhances long‐term interfacial durability.

Altogether, these featuring approaches demonstrate how the all‐in‐one design can evolve from conceptual ideal into practical, scalable solutions for high‐efficiency and durable MEAs. Despite their potential, all‐in‐one MEAs face several challenges, including fabrication complexity, interfacial stability, and trade‐offs between conductivity, mass transport, and mechanical integrity. Scaling precise integration techniques while maintaining performance uniformity remains difficult. Moreover, mismatched material properties can cause interfacial degradation over time. To realize practical applications, future efforts must focus on scalable processing, robust interfaces, and balanced multifunctionality. Furthermore, most reported stability data are limited to short‐term tests (typically < 1000 h) and conducted under idealized conditions. Therefore, we suggest that standardized long‐term durability protocols (e.g., > 2000 h under dynamic operation) should be established to enable meaningful comparison between different MEA designs and accelerate their transition toward commercial viability.

### 5.3 Mechanism Study to Improve Water Electrolysis Performance

In comparison with three‐electrode reactions, the electrochemical reaction process in MEAs is more complex, resulting in a lack of comprehensive studies on the reaction mechanism. Enhancing water electrolysis performance in MEAs requires a multi‐faceted approach that integrates catalyst optimization, efficient mass and ion transport, structural stability, and degradation mitigation strategies. Gaining a thorough understanding of the reaction mechanism is highly desirable. In this regard, operando characterization techniques and visualization methods will be effective tools for future studies, as they can identify the true active sites and observe gas/liquid transport processes in real time, thereby guiding the design of high‐performance MEAs for water electrolyzers.

### 5.4 Advanced Modeling and Intelligent Control for MEAs in Water Electrolyzers

The integration of advanced modeling techniques and intelligent control strategies is becoming a transformative approach to optimize the performance, durability, and efficiency of MEAs in water electrolyzers. This trend is driven by the need to manage complex multi‐physics interactions within MEAs and to adapt to dynamic operational conditions, especially when coupled with renewable energy sources. For example, machine learning and artificial intelligence algorithms, combined with big data analysis, are used to predict MEA performance and lifespan and to optimize material design and operating parameters. Digital twin technology is employed to construct a virtual model of the water electrolysis system, enabling online monitoring, fault prediction, and performance optimization. Additionally, multiphysics coupled simulation is used to integrate electrochemical, fluid dynamics, and thermodynamic models, accurately simulating the complex coupled effects within the electrolyzer.

### 5.5 Sustainability and Cost Reduction

As the demand for green hydrogen increases, there will be a greater push toward improving the cost‐effectiveness and sustainability of MEAs production. This includes using lower‐cost materials and more energy‐efficient manufacturing methods. For example, research will focus on developing low‐cost, earth‐abundant catalysts to reduce dependency on precious metals like platinum and iridium. Advanced membrane materials with higher chemical stability and conductivity will also be a priority.

In conclusion, hierarchical structures within MEAs present significant opportunities to push the boundaries of water electrolysis technology, leading to more efficient and cost‐effective hydrogen production. Continued innovation and research will be critical in realizing the full potential of these advanced MEA designs in future energy systems.

## Conflict of Interest

The authors declare no conflict of interest.
